# Author Correction: Disentangling astroglial physiology with a realistic cell model in silico

**DOI:** 10.1038/s41467-019-12712-6

**Published:** 2019-11-04

**Authors:** Leonid P. Savtchenko, Lucie Bard, Thomas P. Jensen, James P. Reynolds, Igor Kraev, Nikolay Medvedev, Michael G. Stewart, Christian Henneberger, Dmitri A. Rusakov

**Affiliations:** 1 UCL Institute of Neurology, University College London, London, WC1N 3BG UK; 2 The Open University, Milton Keynes, MK7 6AA UK; 3 German Center of Neurodegenerative Diseases (DZNE), Bonn, 53127 Germany; 4 Institute of Cellular Neurosciences, University of Bonn Medical School, Bonn, 53127 Germany

**Keywords:** Cellular neuroscience, Biophysical models, Astrocyte

Correction to: *Nature Communications* 10.1038/s41467-018-05896-w, published online 3 September 2018.

The original version of this Article contained two errors in the author affiliations. The affiliation of Michael G. Stewart incorrectly read ‘Institute of Cellular Neurosciences, University of Bonn, Bonn, 53105, Germany’. The correct affiliation of Michael G. Stewart is: ‘The Open University, Milton Keynes, MK7 6AA, UK’.

The affiliation of Christian Henneberger incorrectly read

‘UCL Institute of Neurology, University College London, London, WC1N 3BG, UK

Institute of Cellular Neurosciences, University of Bonn, Bonn, 53105, Germany

German Center of Neurodegenerative Diseases (DZNE), Bonn, 53127, Germany’.

The correct affiliation of Christian Henneberger is:

‘UCL Institute of Neurology, University College London, London, WC1N 3BG, UK

German Center of Neurodegenerative Diseases (DZNE), Bonn, 53127, Germany

Institute of Cellular Neurosciences, University of Bonn Medical School, Bonn, 53127, Germany’

This has now been corrected in both the PDF and HTML versions of the Article.

