# Supplementary material for: Disentangling astroglial physiology with a realistic cell model in silico
Source: Nat Commun. 2018 Sep 3;9:3554. doi: 10.1038/s41467-018-05896-w (PMC6120909; doi:10.1038/s41467-018-05896-w)
Supplement: Supplementary file 1 — Supplementary Information [file 41467_2018_5896_MOESM1_ESM.pdf]

Astrocyte *in silico*

by Savtchenko et al.

SUPPLEMENTARY INFORMATION

SUPPLEMENTARY FIGURES

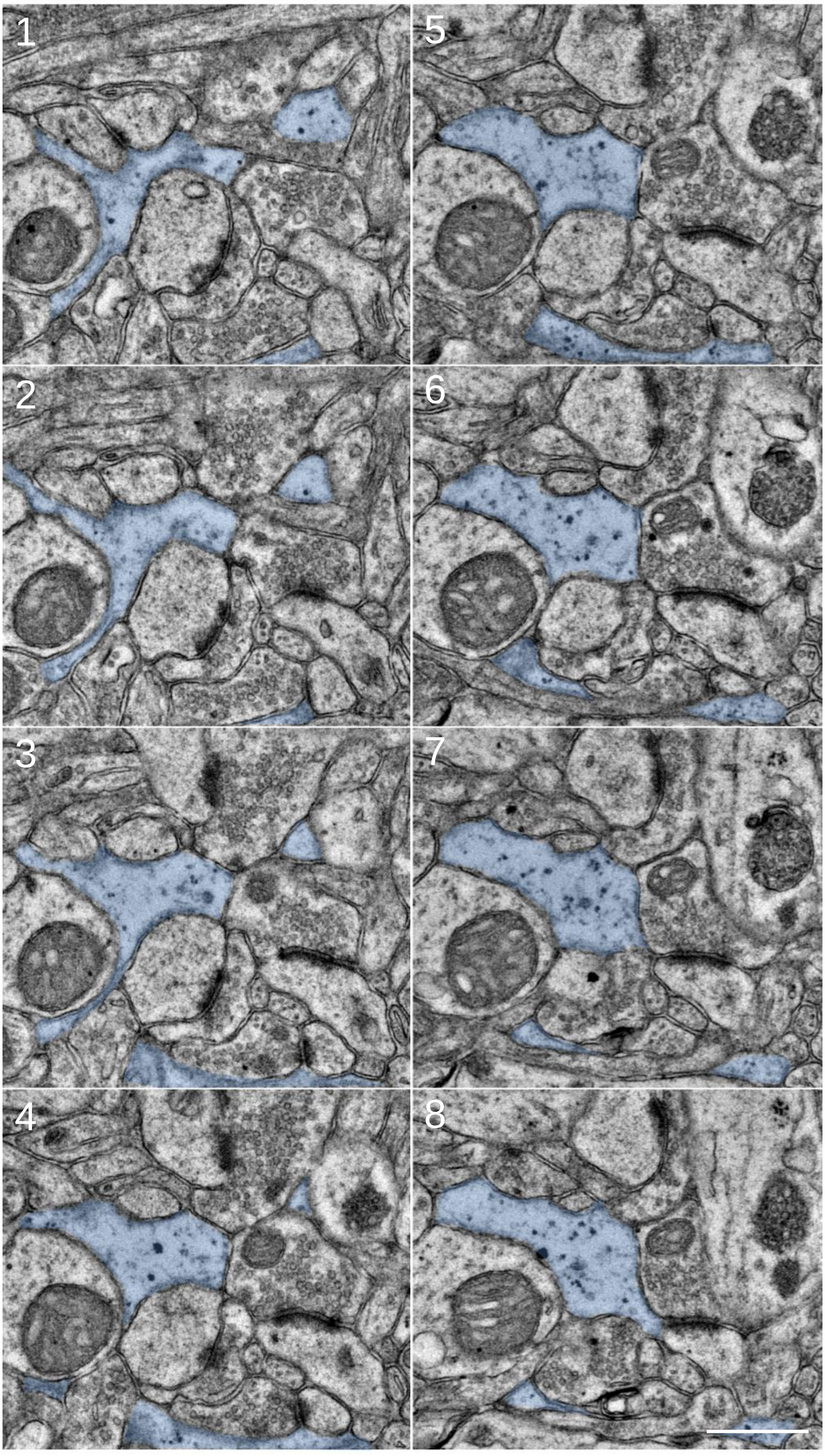

Supplementary Figure 1. An example of serial EM sections with precipitation-labelled astroglia.

Eight (1-8) 60 nm thick adjacent serial sections are shown, as indicated, with areas depicting excitatory synapses and astroglia (blue), filled with electron dense particles resulting from DAB conversion of the recorded astrocyte filled with biocytine. Scale bar, 1 µm.

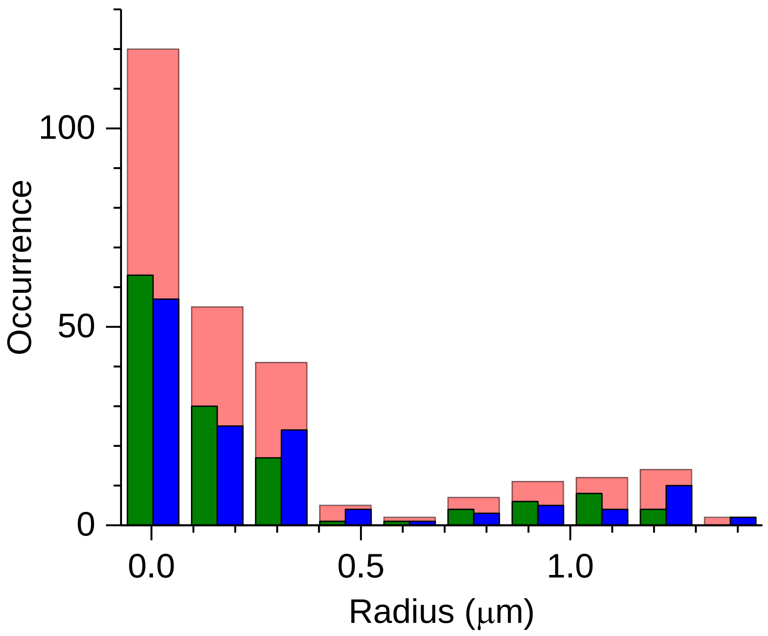

Supplementary Figure 2. Distribution of the cylinder compartment (serial section) radii obtained from the sample of 3D-reconstructed nanoscopic processes of astroglia in area CA1 (*stratum radiatum*) of the hippocampus.

Green, blue, and red column depict transitional, regular, and total (the sum) compartments, respectively (see Fig. 2d,e for detail).

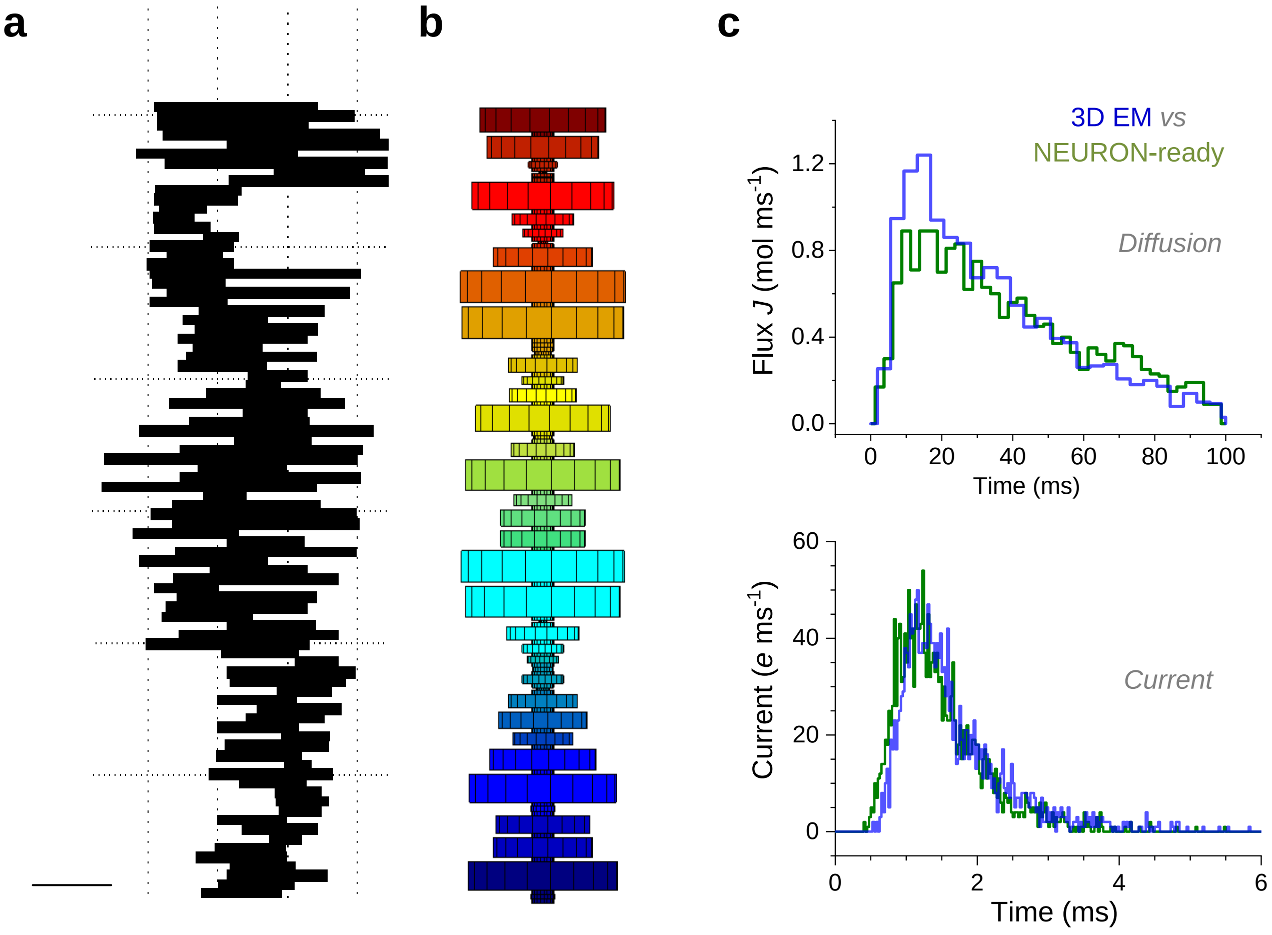

Supplementary Figure 3. Testing biophysical properties of NEURON-based approximations representing nanoscopic astroglial processes.

a, Astroglial process represented by a stack of (60 nm) serial sections based on 3D EM reconstruction. Scale bar, 1 µm.

b, NEURON-compatible representation by a stack of (coaxial) cylindrical sections, with the cylinder diameter distribution matching that of average diameters in the serial section reconstruction as in (**a**).

c, *Top***,** the dynamics (flux) of 2000 Brownian Monte Carlo particles injected at the top of the structures in (**a-b**) and recorded at the bottom of stricture in (**a-b**), as indicated. Bottom, electric current (single charge per millisecond) injected at the top of the structures in (**a-b**) (2000 charges, longitudinal z-direction electric field 10^4 V m^{-1}) and counted at the bottom of the structures in (**a-b**), as indicated.

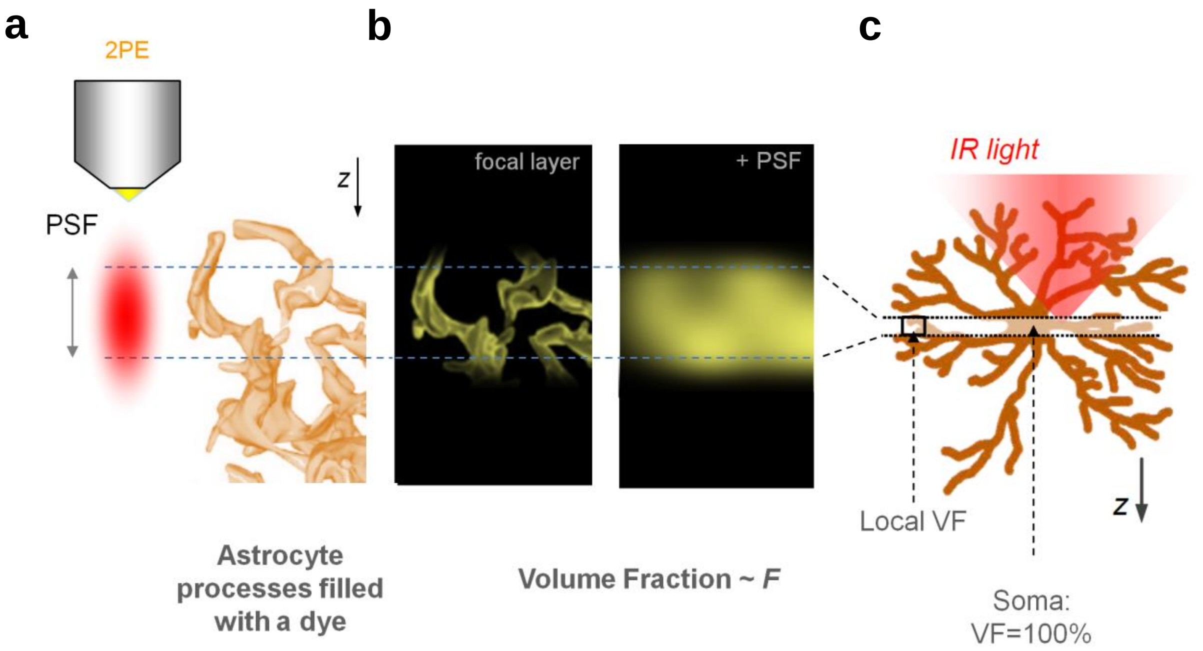

Supplementary Figure 4. Measuring tissue volume fraction (VF) of astroglia.

a, Diagram illustrating the typical point-spread function (PSF, red shade) of two-photon excitation, a dye-filled astrocyte fragment (yellow, 3D EM reconstruction modified from [1]), and the corresponding ~1 µm thick two-photon excitation layer in the focal plane (dotted lines).

b, Intracellular indicator is excited within the focal layer (dotted lines, *left*) and the resulting image is blurred due to the light diffraction determined by the PSF (*right*).

c, On the macroscale, intracellular fluorescence intensity within the 2PE plane (dotted lines) scales with astrocyte tissue volume fraction (local VF), with VF reaching 100% when the plane crosses the soma (5-7 µm wide).

Figure panels a-b were modified from [2].

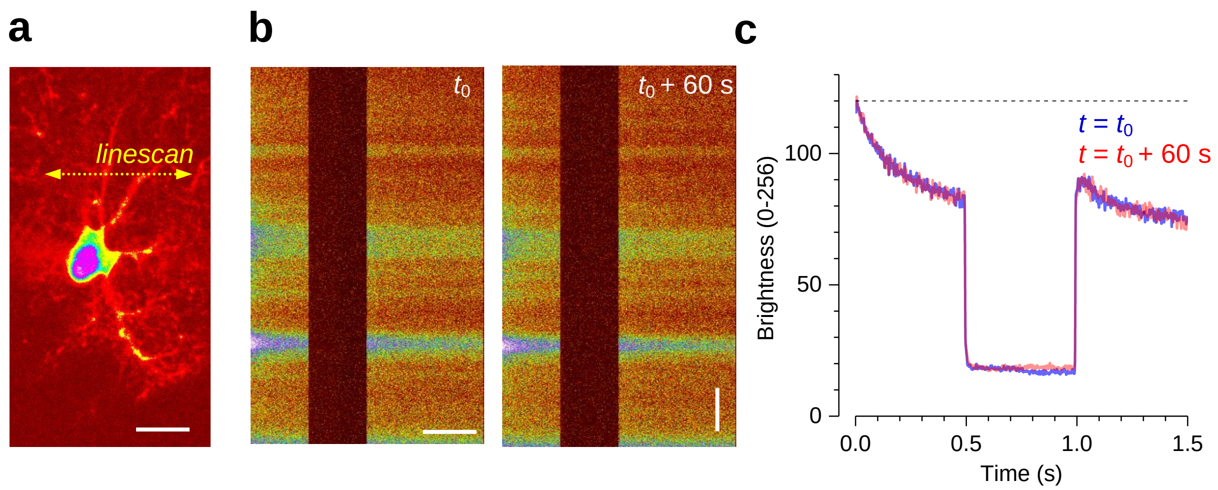

Supplementary Figure 5. Full fluorescence recovery after photobleaching (FRAP) of intracellular Alexa 594 can be seen within 60 seconds.

a, An example of recorded astroglia, linescan position shown. Scale bar, 5 µm.

b, Linescans depicting the photobleaching (between 0-0.5 s), and partial recovery (after the 0.5-1 s shutter-on phase): plot on the right recorded 60 s after the one on the left shows the same brightness kinetics implying full FRAP within <60 s. Scale bar (h, v): 0.5 s, 2 µm.

c, Fluorescence time course for the linescans shown in **b**, as indicated.

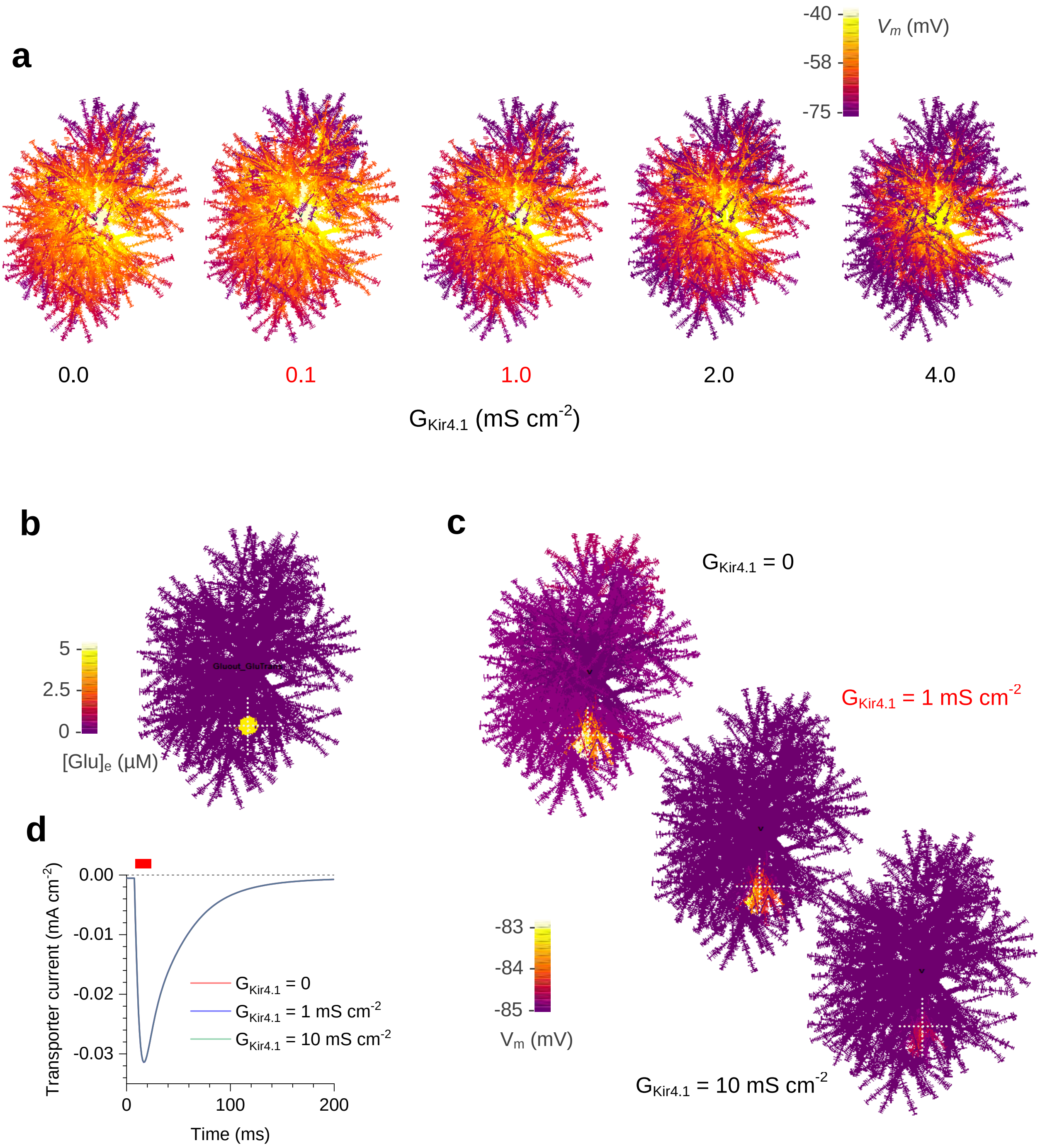

Supplementary Figure 6. Adding potassium channel Kir4.1 conductance within its physiological range has a modest effect on the passive voltage signal transfer or local glutamate uptake current in astroglia.

a, Cell shape diagrams depicting V_m landscape snapshots generated by somatic depolarisation (sine voltage signal) from -75 to -40 mV, with the Kir4.1 channel membrane unit conductance added, at different values as indicated; false colour

scale. The range 0.1-1.0 mS m^{-2} (shown in red) reflects experimental estimates [3, 4]; the default membrane leak conductance is set at 1 mS cm^{-2}.

b, Cell shape diagram showing extracellular concentration of glutamate spot-uncaged at peak concentration $[Glu]_e$ = 10 mM, uncaging spot radius 3 µm (centre shown by crosshair), duration 15 ms; false colour scale.

c, Cell shape diagrams illustrating membrane voltage landscapes at the peak of local glutamate transporter current induced by uncaging, with Kir4.1 channel unit conductance $G_{Kir4.1}$ set at different values, as indicated. The value of 1 mS cm^{-2} (red) is the expected physiological range; baseline membrane leak is set at 1 mS cm^{-2}.

d, Glutamate transporter current kinetics are indistinguishable between the three cases shown in **c**, as indicated; red segment, glutamate uncaging (onset at 5 ms); glutamate transporter GLT-1 kinetics are in accordance with [5].

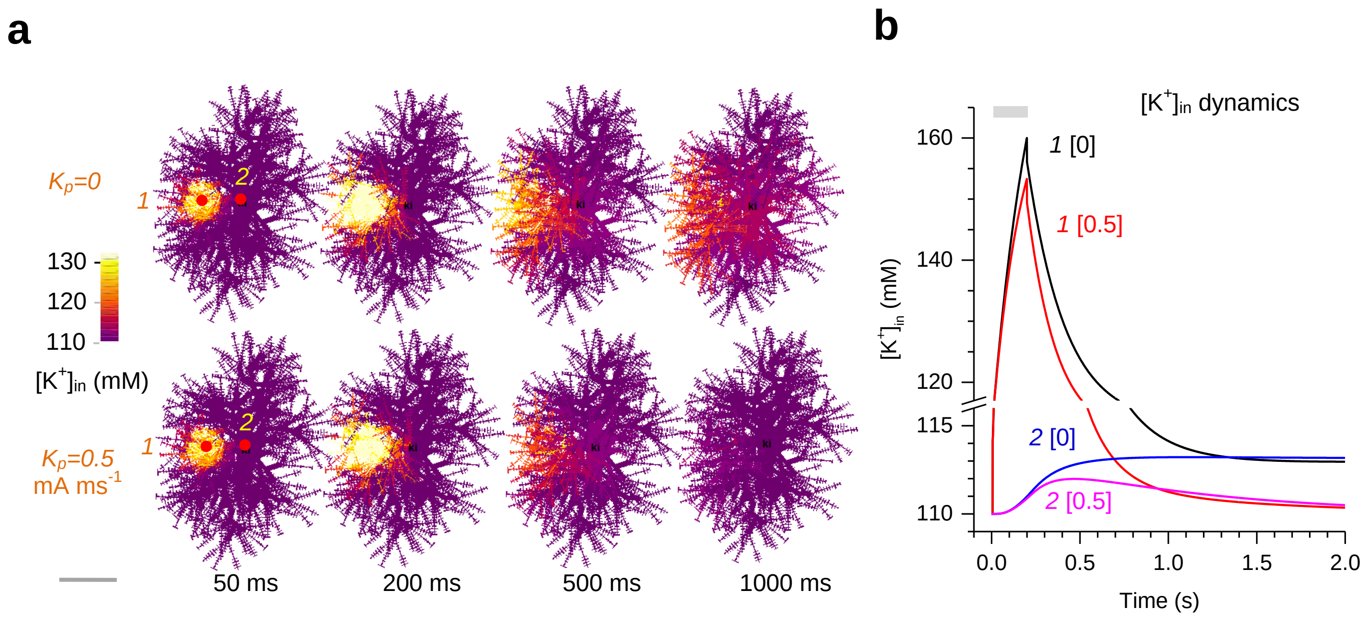

Supplementary Figure 7. Simulated dynamics of intracellular potassium, with and without active extrusion (pump) mechanism.

a, Snapshots of simulated $[K^+]_{in}$ landscapes at different time points following 200 ms long K^+ influx over a ~15 µm area (highlighted in the leftmost diagrams), as indicated (false colour scale). The $[K^+]_{in}$ dynamics was computed in accord with $\frac{d[K^+]_{in}}{dt} = \frac{\pi D}{F}(I_K - K_p)$ where pump current $I_K = K_p\left(\frac{[K^+]_{in}}{[K^+]_{in}^0} - 1\right)$, $[K^+]_{in}^0$ denotes resting $[K^+]_{in}$, F is Faraday's constant, D cell process diameter, K_p is the pumping rate constant (mA cm^{-1}); upper and lower row, K^+ extrusion mechanism off (K_p = 0) and on (K_p = 0.5 mA cm^{-1}; rate compatible with the reported $K_{ir}4.1$ current rate); red dots (marked 1 and 2), points for $[K^+]$ time course recoding shown in **b**. Scale bar, 20 µm.

b, Examples of $[K^+]$ time course (model points 1 and 2 in **a**), with K^+ extrusion mechanisms on ([0.5]) and off ([0]), as indicated.

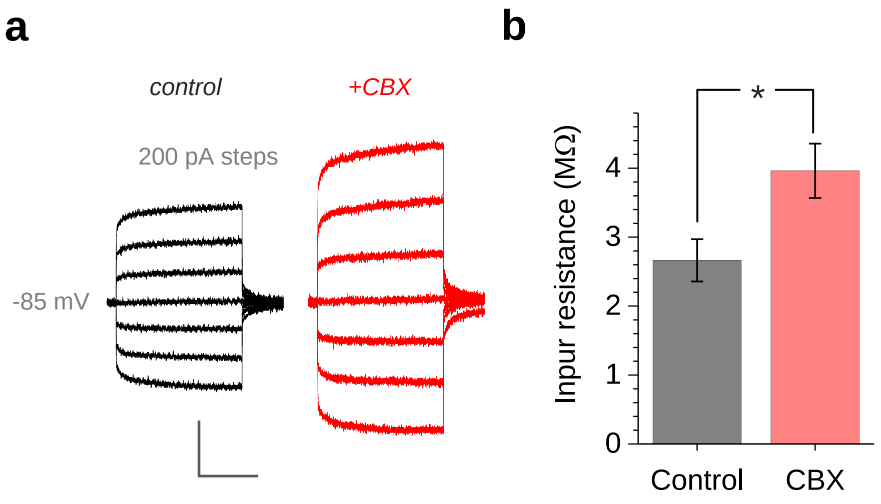

Supplementary Figure 8. Gap junctions and hemichannels contribute ~30% to the basal membrane conductance of CA1 astroglia.

a, Traces show characteristic voltage responses to current steps in CA1 astroglia, in control conditions (black, see also Fig. 4a) and in 50 µm carbonoxelon (red), as indicated. Scale bars (v, h): 1 mV, 250 ms.

b, Bar graphs, average input resistance of CA astrocytes, in control conditions (black, as in Fig. 4a) and in 50 µM carbonoxelon (red); mean ± SEM: 2.66 ± 0.31, and 3.96 ± 0.39 MΩ, respectively; n = 15 and n = 18 for control and CBX groups, respectively; * p = 0.022).

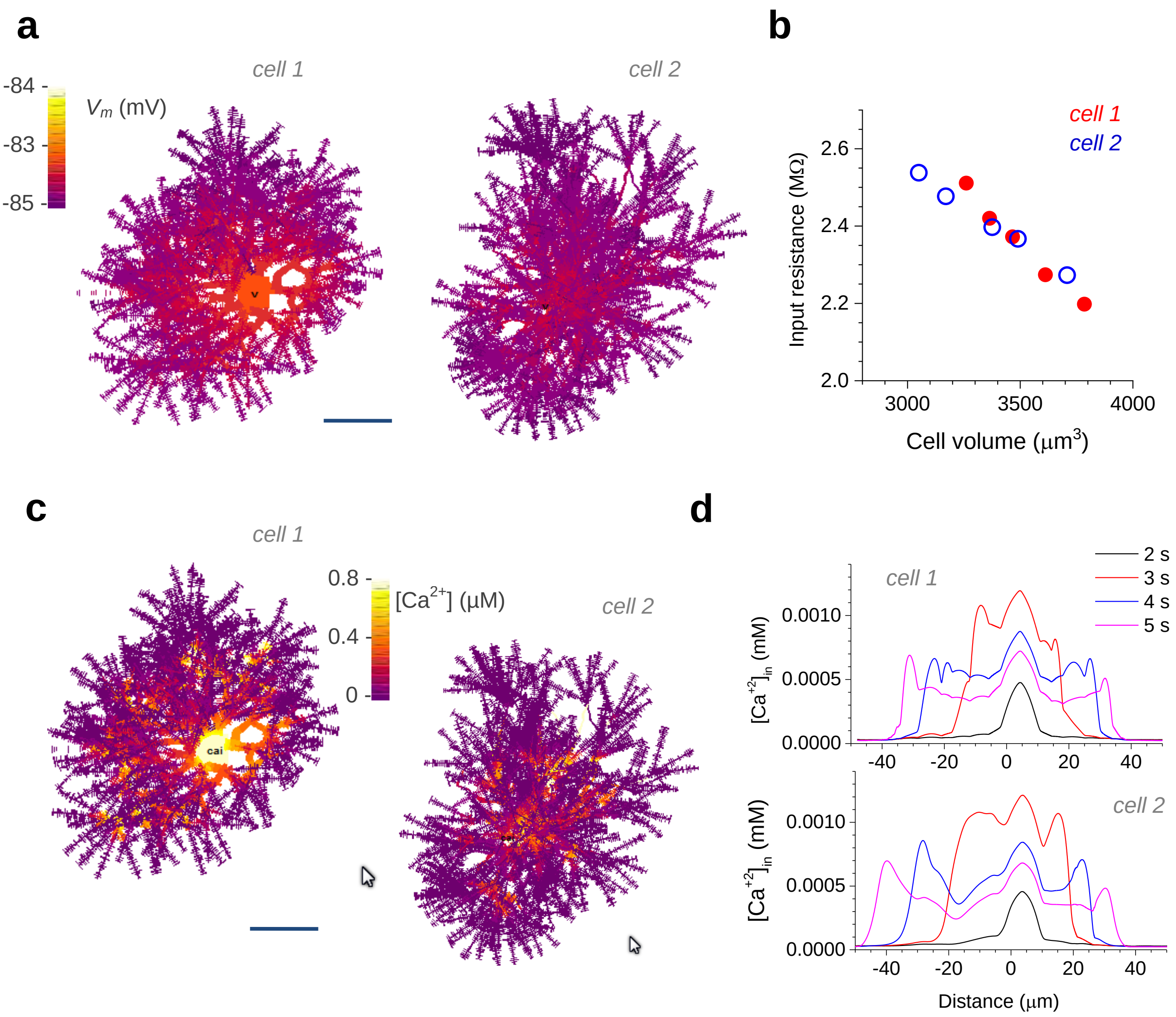

Supplementary Figure 9. Comparing key biophysical traits between two astroglia that possess similar nanoscopic properties but different 'stem trees'.

a, Snapshots of the **v**oltage landscapes generated by a 100 pA current injection at the soma; *cell 1*, cell tree morphology generated by adjusting the library neurogliaform 'stem tree' to the average properties of CA1 astroglia (Fig. 1e); *cell 2*, cell tree morphology 3D-reconstructed from two-photon excitation imaging of an individual CA1 astrocyte (Fig. 1a-d); nanoscopic processes of both cells are generated using the same statistics for process seeding, geometry and tissue volume fraction; cell 2 soma is partly obscured by cell branches in 3D. Scale bar, 20 μm.

b, Input resistance values for cell 1 (red) and cell 2 (blue) across a sample of five stochastically generated nanoscopic-process morphologies under fixed stem trees, in each cell; membrane unit conductance, 1 mS cm^{-2}.

c, Snapshots of [Ca^{2+}] landscape 3 s after Ca^{2+} wave triggering by IP_3 release (2.5 µM step, onset at 1s; cell 2 soma is partly obscured by cell branches in 3D). Relevant simulation parameters: Ca^{2+} diffusion coefficient, 0.3 µm^2 cm^{-1}; immobile Ca^{2+} buffer concentration, 200 µM (K_f = 25 mM^{-1} ms^{-1}; K_D = 2 ms^{-1}); mobile Ca^{2+} buffer concentration, 10 µM (K_f = 600 mM^{-1} m^{-1}; K_D = 0.5 ms^{-1}; diffusion coefficient 0.05 µm^2 cm^{-2}); Ca^{2+} pump activation threshold, 50 nM; Ca^{2+} pump flux density, 20 µM cm^{-1}; basal IP_3 concentration, 0.8 µM; see main text and User Guide text for further detail and parameter explanations. Scale bar, 20 µm.

d, Dynamics of [Ca^{2+}] profile (crossing the soma) during wave propagation in cell 1 and 2, as indicated; time points, interval following the wave triggering onset.

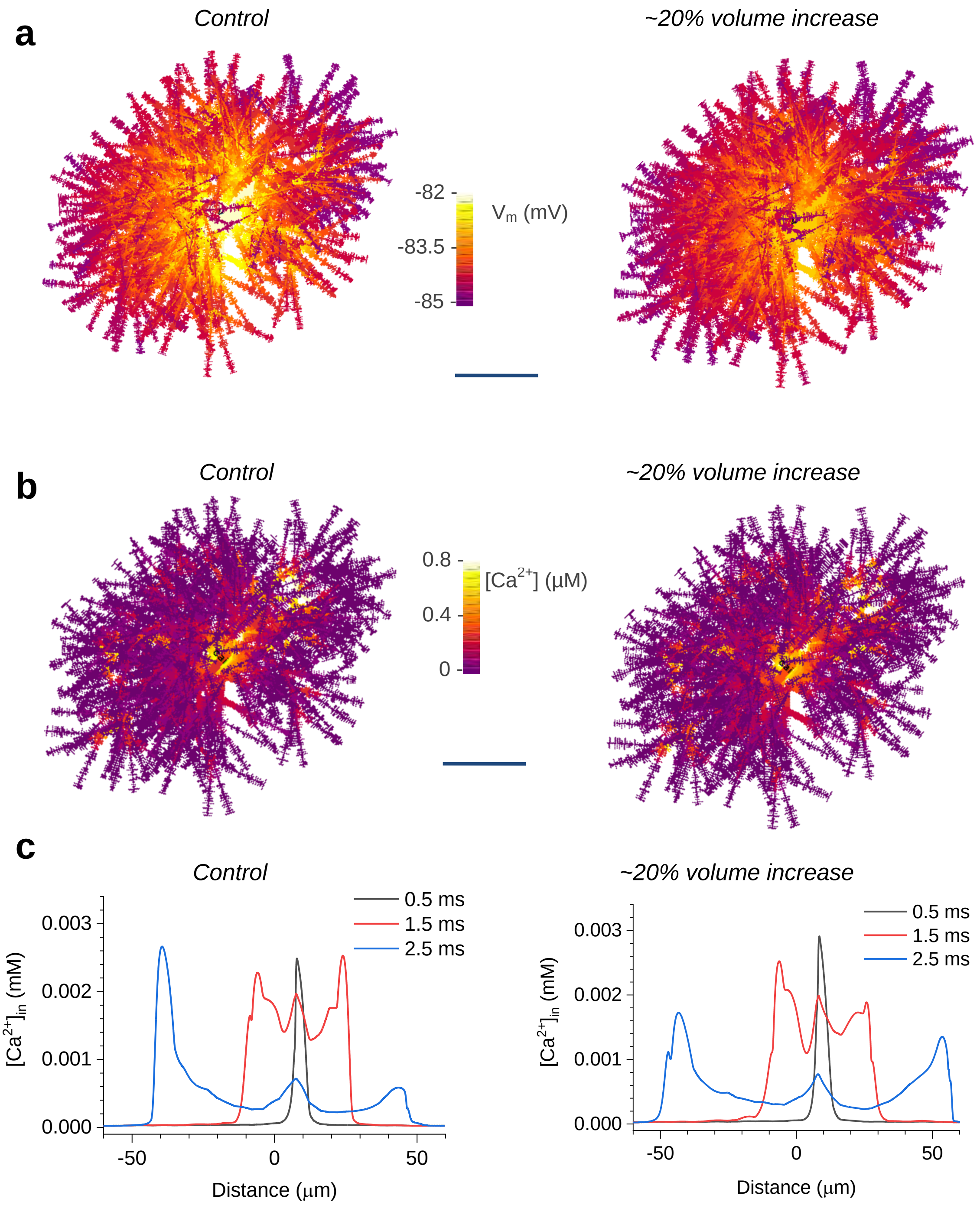

Supplementary Figure 10. The effect of cell volume change on the membrane voltage landscape and Ca^{2+} wave propagation in astroglia.

a, Snapshots of the voltage landscapes generated by a 1 nA current injection at the soma in the 'Control' astrocyte model (as elsewhere in the study) and in the same astrocyte model but with the cell compartment cross-section areas increased by ~20% throughout, as indicated. Scale bar, 20 µm.

b, Snapshots of [Ca^{2+}] landscape 1 s after Ca^{2+} wave onset triggered by IP_3 release (2.5 µM step) in two cells depicted in **a**. Relevant simulation parameters: Ca^{2+} diffusion coefficient, 0.3 μm^2 cm^{-1}; immobile Ca^{2+} buffer concentration, 200 µM (K_f = 25 mM^{-1} ms^{-1}; K_D = 2 ms^{-1}); mobile Ca^{2+} buffer concentration, 10 µM (K_f = 600 mM^{-1} m^{-1}; K_D = 0.5 ms^{-1}; diffusion coefficient 0.05 μm^2 cm^{-1}); Ca^{2+} pump activation threshold, 50 nM; Ca^{2+} pump flux density, 20 µM cm^{-1}; basal IP_3 concentration, 0.8 µM; see main text and User Guide text for further detail and parameter explanations. Scale bar, 20 µm.

c, Dynamics of [Ca^{2+}] profile (crossing the soma) during wave propagation in cells depicted in **b**, as indicated; time points, interval following the wave triggering onset.

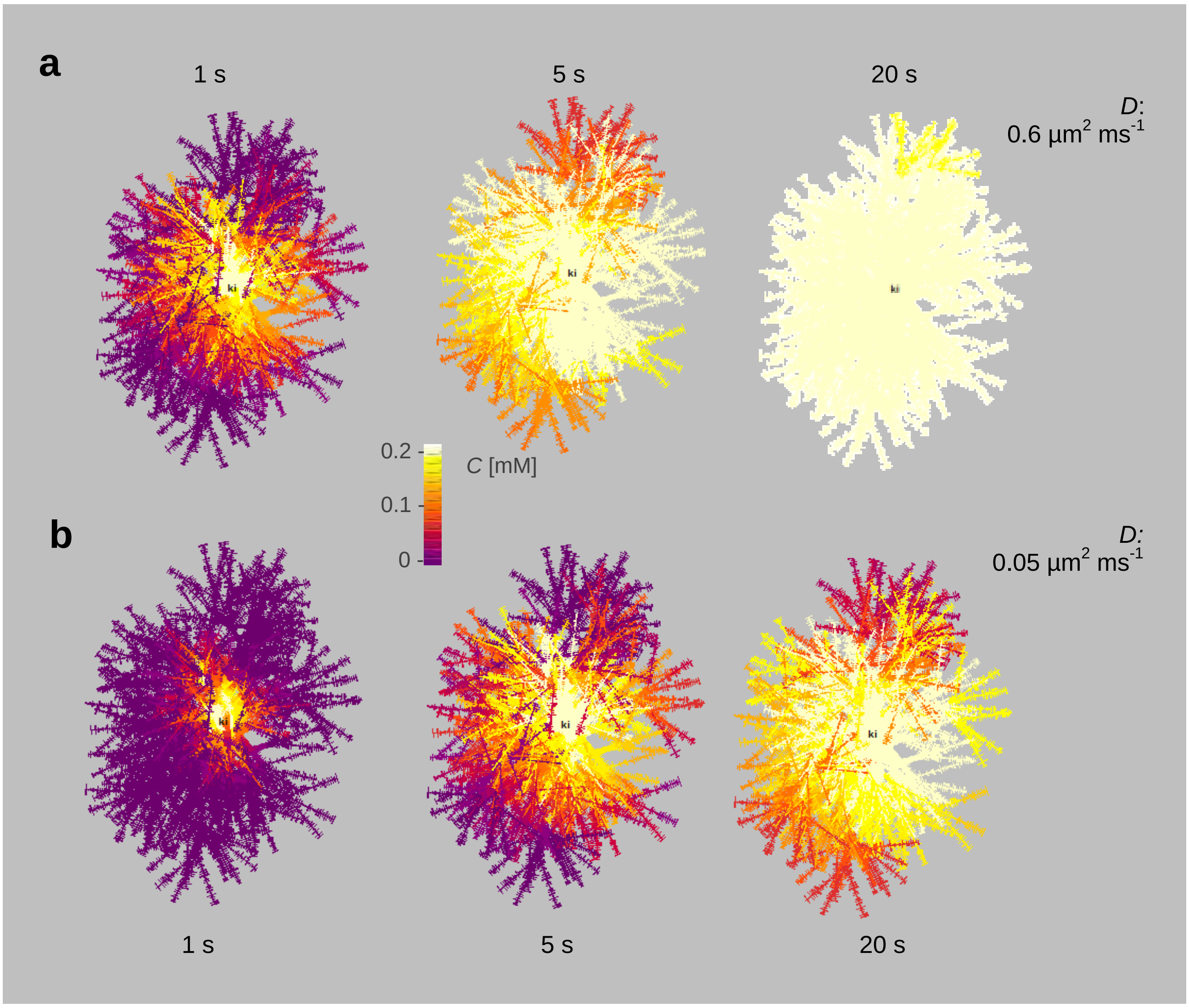

Supplementary Figure 11. Diffusion equilibration inside astroglia depends strongly on the molecule diffusion coefficient.

a, Snapshots of the intracellular concentration landscape at different times after 'patching' the soma with 200 µM concentration-clamped diffusion source, as indicated; D = 0.6 µm^2 ms^{-1} corresponds to diffusivity of small ions.

b, Same as in **a** but with D = 0.05 µm^2 ms^{-1}, which corresponds to obstacle-free diffusivity of larger molecules such as Alexa-dextran (MW ~3kDa). Note that in real experiments larger molecules are likely to undergo significant steric and viscous hindrance in the cytoplasm of nanoscopic cell compartments, which could slow down their diffusion disproportionally. The effective diffusion coefficient (free parameter) has to be established by matching simulation outcome to the empirical data.

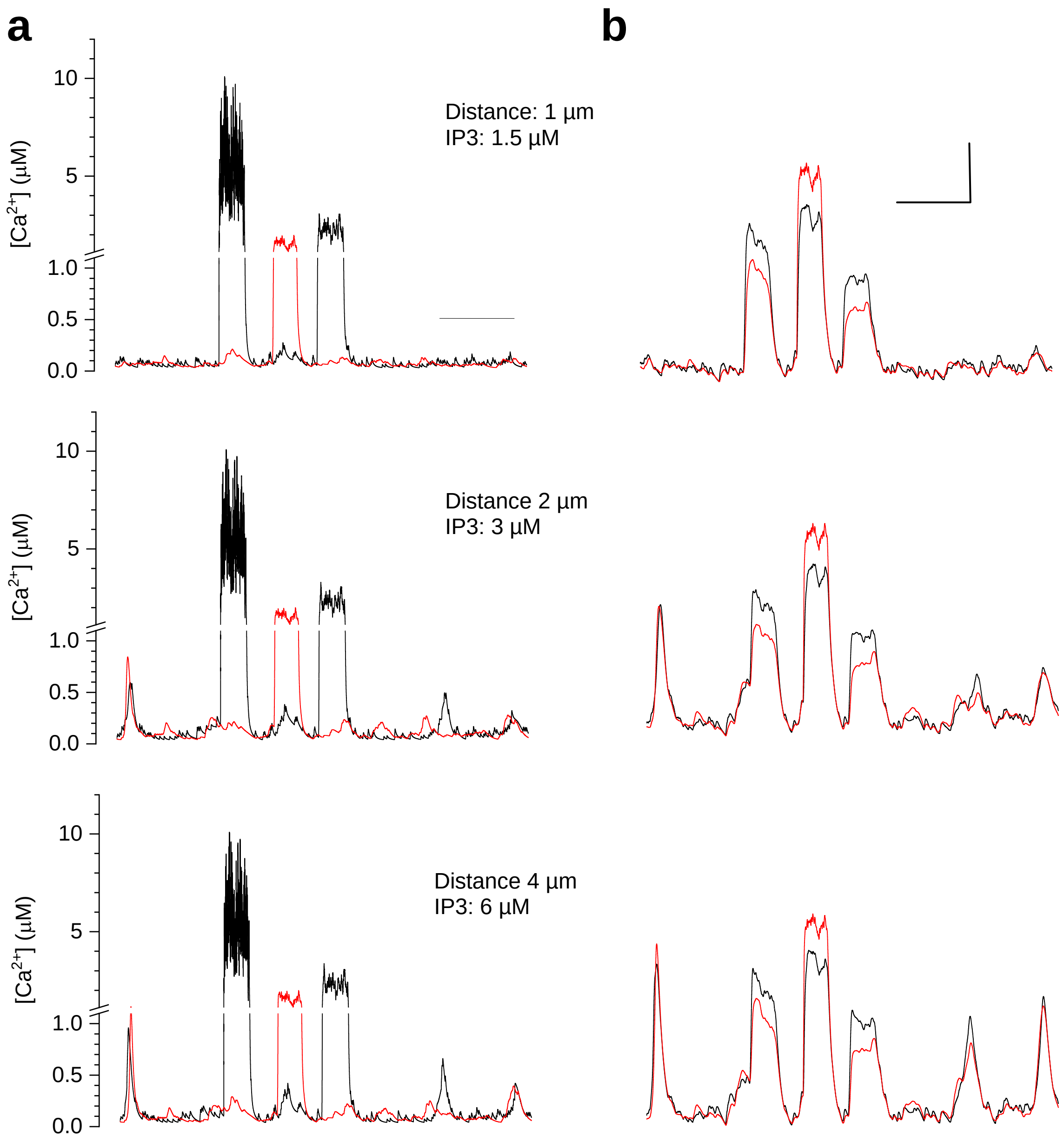

Supplementary Figure 12. Intracellular [Ca^{2+}] dynamics, and its fluorescent readout, in astrocyte branches under varied clustering of intracellular IP_3 receptor activity.

a, Simulated intracellular [Ca^{2+}] dynamics: branch 1 (black) and branch 2 (red) correspond to loci 1 and 3, respectively, on cell diagram shown in Fig. 7c. Parameters of Ca^{2+} homeostasis and IP_3 dependent activity are set as in the Fig. 7 simulations, but at different clustering arrangements for IP_3 receptor activation, as indicated (1, 2, and 4 μm), with the local peak IP_3 levels (reflecting receptor activation) adjusted to

keep the total unchanged (1.5, 3, and 6 µM, as indicated). Note two-scale ordinate. Scale bar, 5 s.

b, Time course of simulated Ca^{2+}-dependent fluorescent signal (proportional to Ca-bound 150 µM Fluo-4 'added' to the cytoplasm) that corresponds to the [Ca^{2+}] dynamics shown in **a**. Note how indicator saturation masks large differences in the underlying free [Ca^{2+}] transients. Scale bards (v, h): 0.2$\Delta F/F$, 5 s.

SUPPLEMENTARY TABLE

Supplementary Table 1. Basic morphological parameters of modelled astroglia

	**'Typical-tree' astrocyte[1]**	**'3D reconstructed-tree' astrocyte[2]**	**'Library stem-tree' astrocyte[3]**
No of all main branches	131	145	125
1st order branches	8	10	10
2nd order branches	33	27	19
Main Branch diameter<br><br>Mean±SD, µm<br>min, µm<br>max, µm<br>median, µm	 <br>1.42±0.53<br>0.834<br>3.61<br>1.28	 <br>1.02±0.69<br>0.182<br>5.45<br>0.927	 <br>1.42±0.55<br>0.83<br>3.60<br>1.27
Total length of identifiable (main) branches, µm	763	999	628
Mean volume fraction (between soma-30 µm)	0.066±0.035	0.0501±0.037	0.0562±0.049
Leaf diameter Mean±SD, µm	1.407±0.663	1.2247±0.62	1.17±0.72
Stalk diameter Mean±SD, µm	0.2±0.15	0.367 ±0.3619	0.2±0.15
Nanoscopic process length Mean±SD, µm	13.5 ±2.5	13.5 ±2.5	13.5 ±2.5
No of nanoscopic processes	260	288	248

Cell volume[4]<br>Mean±SD, μm^3<br><br>with soma<br>without soma	 <br><br>3280±528<br>2094±291	 <br><br>2506±520<br>1668±250	 <br><br>4190±310<br>1910±194
Cell surface area[4]<br>Mean±SD, μm^2<br><br>with soma<br>without soma	 <br><br>45260±10327<br>42723±8700	 <br><br>45860±8700<br>41423±7600	 <br><br>41580±8200<br>35815±8410
Surafe/Volume ratio[4]<br>Mean±SD, μm^{-1}<br><br>with soma<br>without soma	 <br><br>13.78±1.10<br>20.38±2.14	 <br><br>18.3±2.69<br>24.83±0.72	 <br><br>11.06±1.13<br>18.75±2.26
Total No of compartments[4], Mean±SD	40200±4240	43836±4320	37776±3720

Notes:

[1] Related to Figs. 3, 4, 7; stem-tree file AstrocyteBasicGeometry.hoc

[2] Related to Figs. 1a-c, 5; Supplementary Fig. 9b, d; stem-tree file RealAstrocyteSkeleton1.hoc

[3] Related to Figs 6a; Supplementary Fig. 9a,c; Supplementary Fig 10; stem-tree file GliaFormP32_DEV136.hoc

[4] Varied due to the stochastic nature of trial-to-trial generation of nanoscopic processes.

SD, standard deviation.

SUPPLEMENTARY NOTE 1

USER GUIDE

ASTRO 1.0.

Astrocyte *in silico*

© University College London, MIT licence

2017

TABLE OF CONTENTS

INTRODUCTION 4
Key system and software requirements 4
Basic version 4
Full version 4
The strategy of building the model: summary 5
GETTING STARTED 7
Installing and running ASTRO 7
Setting up and launching 7
Setting up and running ASTRO: Main modules 9
Setting up and running ASTRO: Worker (cluster) computer preparation 10
ADOPTING EMPIRICAL DATA ON ASTROCYTE MORPHOLOGY 10
Main cell processes and branches 10
Generating nanoscopic processes of astroglia 11
Uploading a 3D-reconstructed fragment of astroglia 11
Storing a representative sample of 3D-reconstructed nanoscopic processes 12
Transforming 3D-reconstructed processes into sets of disk-shaped compartments 14
Biophysical comparison of original and NEURON-compatible structures: Monte Carlo test settings 15
Biophysical comparison of original and NEURON-compatible structures: running the test 16
Statistics of NEURON-compatible cylindrical compartments 18
GENERATING COMPLETE ASTROCYTE MORPHOLOGY 19
File structure in Host computer (under Windows) 19
Preparing ASTRO system files 19
Generating astrocyte stem tree (including endfoot) 20
Generating astroglial morphology on the nanoscale 21
Geometry of nanoscopic processes 21
Populating astrocyte tree with nanoscopic processes 22
Tissue-filling properties of astroglial morphology 23
Generating and exploring pre-determined astrocyte models 24
SIMULATING ASTROGLIAL FUNCTION 24
Linescan FRAP experiment: probing intracellular connectivity of astroglia 24
Probing membrane mechanisms of astroglia 26
Membrane voltage landscape 26

Simulating membrane voltage in response to local current hotspots 27
Gap junctions 27
Modelling intracellular calcium dynamics 28
Ca^{2+} wave simulations 28
Choosing between IP_3-dependent mechanisms of Ca^{2+} signalling 30
Simulating microscopic Ca^{2+} events 30
Simulating astroglial glutamate transporters 31
Simulating potassium dynamics inside and outside astroglia 33
SYSTEM PREPARATIONS FOR HIGH-END CALCIUM SIMULATIONS 35
Preparing Worker computer / cluster (HPC, OS Linux) for Ca^{2+} simulations 35
Preparing Host computer (client, OS Windows) for Ca^{2+} simulation 36
Technical Notes 39
Nano geometry 39
Calcium dynamics (Cluster) 39

INTRODUCTION

Electrically non-excitable astroglia take up neurotransmitters, buffer extracellular K^+ and generate intracellular Ca^{2+} waves which can release molecular regulators of local neural circuitry. The underlying cellular machinery remains enigmatic, mainly because the nanoscopic, sponge-like astrocyte morphology has been difficult to access experimentally and explore theoretically. The simulation tool ASTRO described here helps to evaluate the multi-scale morphology of astroglia and to create a realistic, multi-compartmental biophysical model of an astrocyte that could be explored using the NEURON computational environment. The latter enables a wide range of biophysical cellular mechanisms to be incorporated and tested in the model. Thus, simulations with ASTRO aim to assist with the mechanistic interpretation of experimental observations in astroglia.

Key system and software requirements

The current version of ASTRO can be downloaded directly from https://github.com/LeonidSavtchenko/Astro. The present User Manual is to be regularly updated. Its current version can be downloaded from the same location.

Basic version

Running ASTRO without full-scale simulations of intracellular Ca^{2+} dynamics requires:

1. Host computer must have MATLAB (2012 version or later, https://uk.mathworks.com/products/matlab.html) and NEURON (7.2 or later, https://neuron.yale.edu/neuron/download) installed under Windows 7 or Windows 10 (Windows 8 was not tested).

Full version

Simulating full intracellular Ca^{2+} dynamics on top of other biophysical mechanisms is highly resource-consuming and should normally require:

1. Two computers: the Host computer operating under Windows, and Worker (remote) computer cluster operating under Linux.
2. Basic preinstalled software: MPIC++ (Worker) (https://www.open-mpi.org/software/ompi/v3.0/), MATLAB not older than 2013 (Worker and Host) and NEURON 7.0 (Worker and Host) https://neuron.yale.edu/neuron/download.
3. Platform: Linux and Windows. Optional modes of operation: sequential and parallel (MPI) computing.

NOTE: Instead of the fully-fledged MATLAB version, the user can install MATLAB Runtime, which is a free package that can be downloaded from here: https://www.mathworks.com/products/compiler/mcr/.

The strategy of building the model: summary

The morphology of brain astroglia differs significantly from that of nerve cells, notably in two main aspects. Firstly, it features a complex system of nanoscopic processes that originate from several primary (as well as secondary and some tertiary) processes filling the tissue volume in between the branches. Because the size of these nanoscopic processes is beyond the diffraction limit of conventional optical microscopy (including two-photon excitation imaging), they are normally visualised as a cloudy structure surrounding thicker branches. Secondly, tissue domains occupied by individual astroglia do not overlap: thus, individual cells fill in all the tissue space that corresponds to the cell territory. Finally, astrocytes from different brain regions could have different morphologies and employ different cellular mechanisms. Bearing this in mind, the strategy of building an astrocyte model that recapitulates real astroglia morphology and known astroglial function consists of three main stages (Fig. 1).

Stage 1 is to replicate *in silico* the geometry of all main cell processes (stem tree) that could be identified and traced in an optical microscope. Here the procedure is somewhat similar to well-established routines of neuronal reconstruction including: (i) 3D optical reconstruction of the cell filled in with a fluorescent indicator, in live or fixed tissue; (ii) tracing the soma and all identifiable (thick) branches while cutting off the 'cloudy' area representing nanoscopic processes; and (iii) saving the main-branch 3D geometry (including variable thickness of individual branches) in a NEURON-compatible digitised format. This procedure could be performed using various available software types, such as NEUROLUCIDA or Vaa3D (see sections below for detail). Alternatively, the stem tree could be, first, downloaded from the on-line library or generated computationally, and second, adjusted in accord with the experimental measurements obtained for the astroglia population under study.

Stage 2 (Fig. 1) is to generate *in silico* a complex system of nanoscopic processes that would originate from the thicker branches (reconstructed during the **first stage**) filling the space between them. Because the exact reconstruction, *in situ* or *in silico*, of many hundreds or thousands of ultrathin leaf-like astrocyte processes is not technically feasible, the method adopted here is to generate such branches automatically based on the statistical morphometric data obtained using 3D electron microscopy (EM) and the volumetric data obtained with two-photon excitation imaging. To achieve this, the complex and irregular geometry of 3D-reconstructed nanoscopic processes is transformed into NEURON-compatible shapes involving cylindrical (disk-shaped) compartments. Importantly, this transformation is tested for biophysical compatibility (key electrodynamic and diffusion properties) between the original and NEURON-compatible 3D shapes.

Stage 3 (Fig. 1) is to adjust basic biophysical properties of the modelled astroglia by constraining free (unknown) parameters of the model using the data of electrophysiological or optical/ ligand probing tests *in situ*.

Thus, a realistic cell model that could represent the astrocyte cohort under study will require a set of experimental data, either published separately, accessible through an on-line library, or obtained *de novo*, that are sufficient to carry out the above stages of model construction. The section below explains what type of experimental data are required to build a realistic astrocyte model *de novo* (from scratch) based on one's own observations.

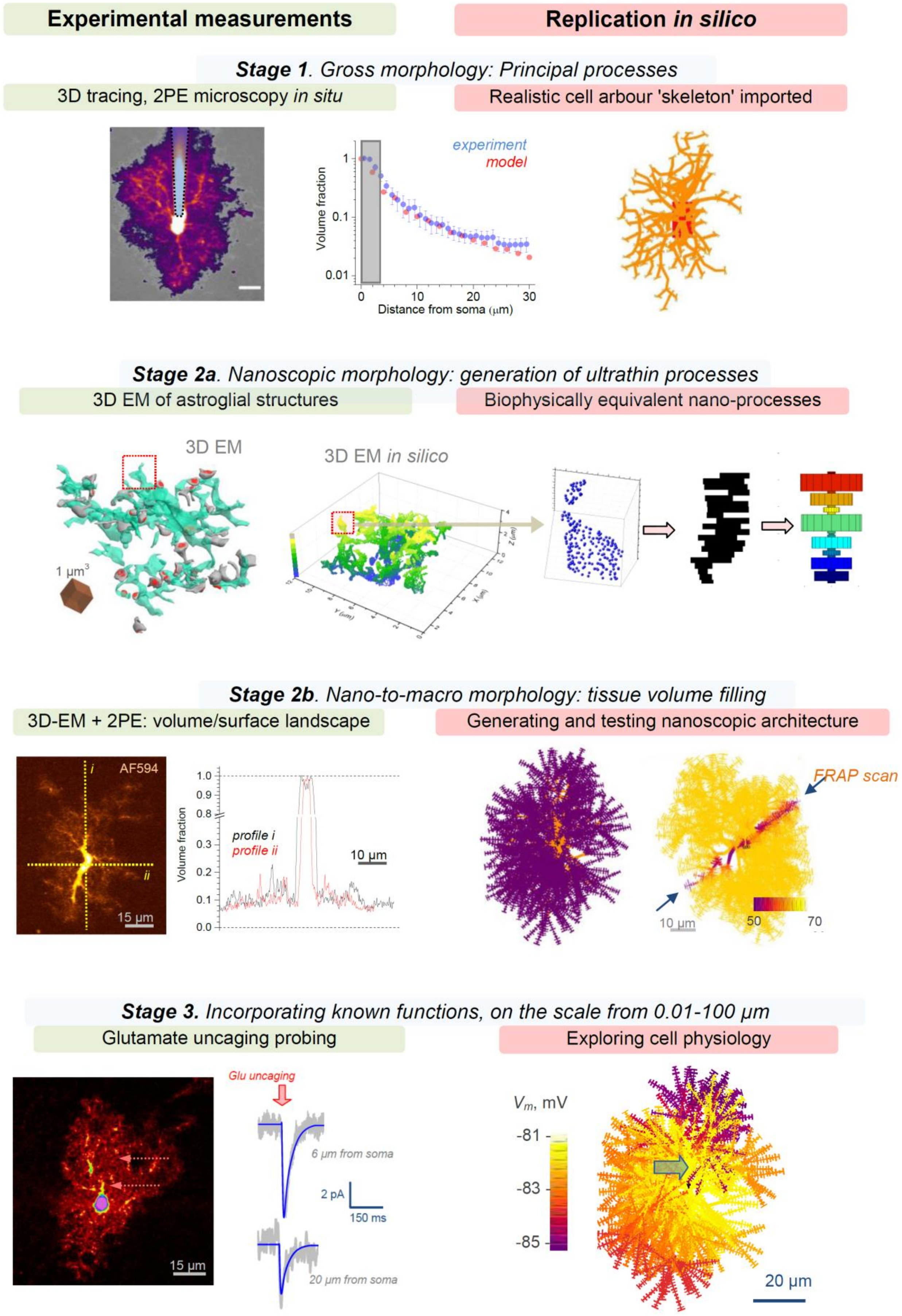

Figure1. Astroglia *in silico*: summary of the modelling approach.

Experimental data required to constrain a de novo realistic astrocyte model

1. A 3D reconstructed tree of main identifiable astroglial processes digitised in a NEURON-compatible format. Alternatively, this could be an artificially generated cell arbour with the branching pattern and branch diameters representing the average (typical) astrocyte from the population of interest.

2. A sample (20-50) of nanoscopic astroglial processes reconstructed using 3D (serial-section) EM, with rendered surface co-ordinates. This sample will be used to obtain statistical properties of the nanoscopic astroglial processes generated by the model.

3. Average tissue volume fraction occupied by astroglia, as distributed radially from the soma to the cell edges. This data set can be obtained from two-photon excitation or 3D EM measurements *in situ*.

4. The mean membrane surface density and the surface-to-volume fraction values obtained from 3D EM reconstructions of nanoscopic astroglial processes.

5. Functional data pertaining to the electrophysiological and biophysical properties of the astroglia of interest, such as the characteristic I-V curves (somatic patch-clamp, square-pulse current injections), electrical responses to glutamate/GABA application or uncaging, data on the ion (K^+, Na^+) homeostasis, documented characteristics of intracellular calcium wave , etc.

GETTING STARTED

Installing and running ASTRO

Setting up and launching

The latest installation version can be downloaded from (https://github.com/LeonidSavtchenko/Astro). On the website front page (Fig. 2a), to download Astro, press the green key **'Clone or download'** and save Download.Zip at any place of the personal computer. Then the archive must be opened and its content saved on the Host computer (Windows/MacOs) keeping the folder structure as described (Fig. 2b).

The full version of ASTRO (which is required for full-scale computations of intracellular Ca^{2+} activity) requires a computer cluster that runs under Linux. See details in the section '**Preparing Worker computer / cluster (HPC, OS Linux) for Ca^{2+} simulations**' and '**Setting up and running ASTRO: Worker computer preparation**'.

To get started with ASTRO in '**Nano (Geometry)**' and **'Simulations (NEURON)'** modules, the Host computer must have MATLAB (2013 or later) and NEURON (7.0 or later) installed. To get started with ASTRO in the **'Simulations (NEURON)'** only, the Host computer must have NEURON (7.0 or later) installed.

Executing ***Start.m*** in MATLAB environment preinstalled on the Host computer generates an introductory menu (Fig. 3).

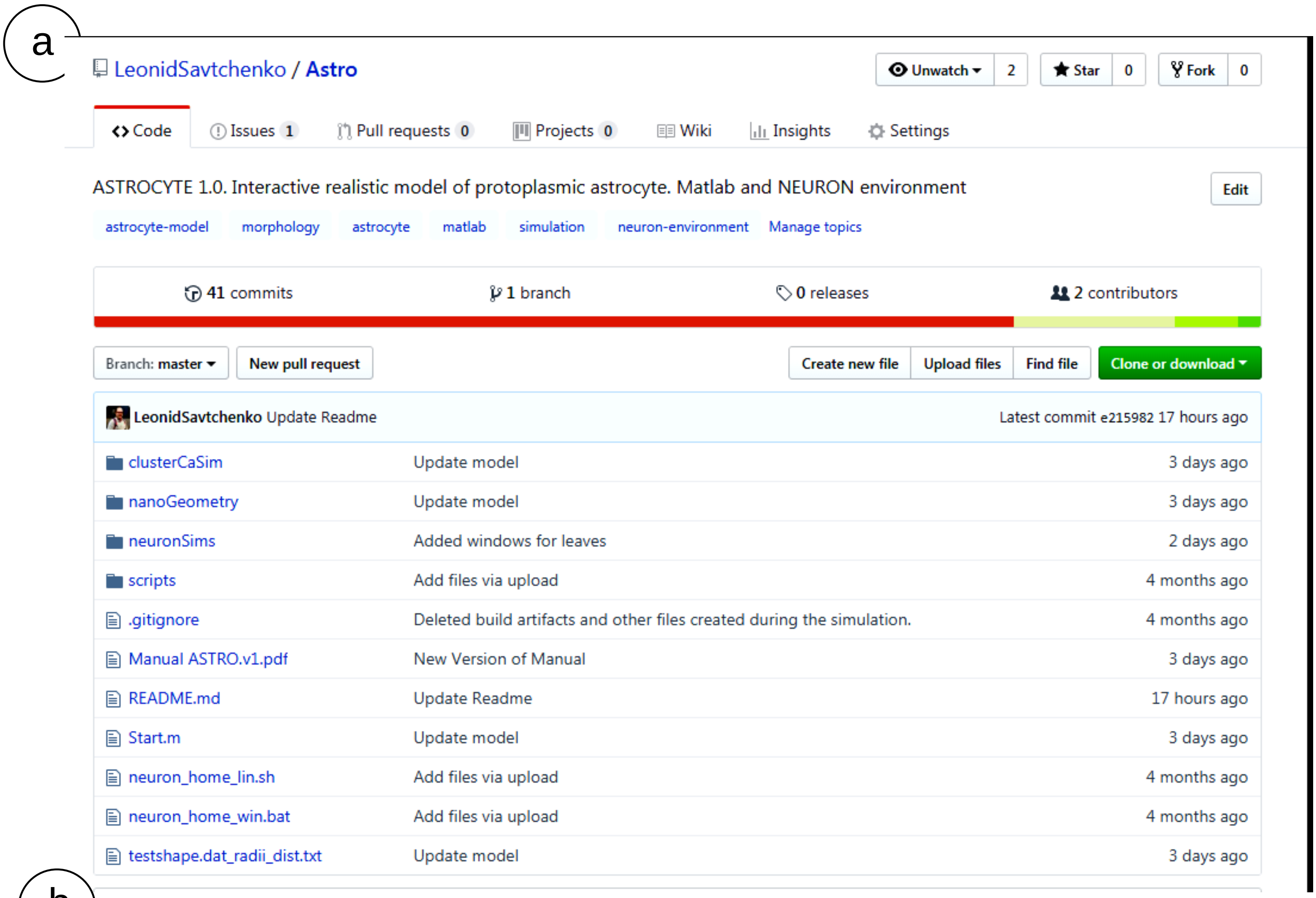

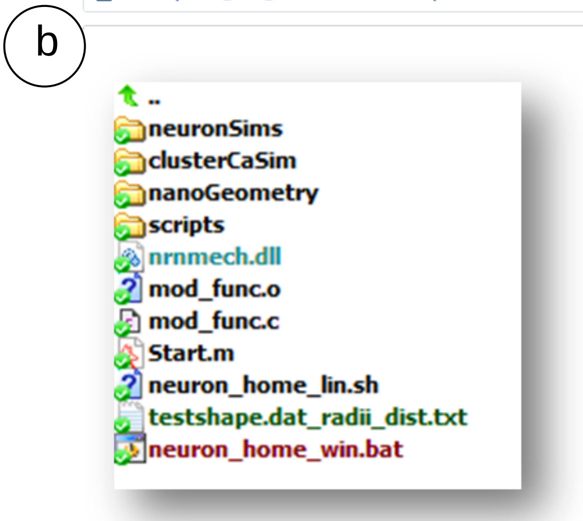

Figure 2. Screenshot of the ASTRO download webpage (a), and folder structure or ASTRO1.0 on the Host computer (b).

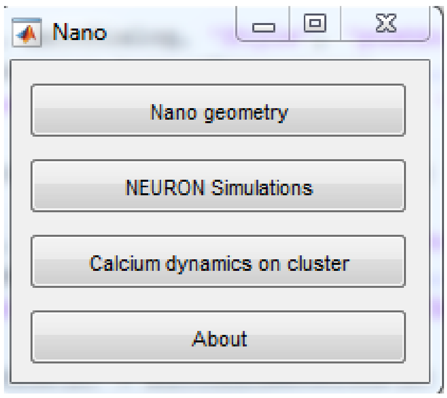

Figure 3. Introductory menu.

The menu provides the choice of the three main simulation modules, **'Nano (Geometry)'**, **'Simulations (NEURON)'**, and '**Calcium dynamics (Cluster)**'. The two former modules can be run routinely on a stand-alone Host computer whereas the '**Calcium dynamics (Cluster)**' module, which deals with resource-consuming simulations of intracellular astroglia Ca^{2+}, requires the Worker computer / cluster, as explained below.

Setting up and running ASTRO: Main modules

1. **'Nano (Geometry)'** focuses on the construction of realistic astroglial morphology on multiple scales. This MATLAB-based module is designed: (a) to import the 3D main-branch (stem tree) morphology of astroglia in a NEURON-compatible format; (b) to import a 3D-EM reconstructed astroglial fragments and to analyse geometry of the nanoscopic processes therein; (c) to populate astroglial stem tree with nanoscopic astroglial protrusions that reflect experimental 3D-EM data. This module can be run separately from other parts of ASTRO, it requires a Host computer with MATLAB (2013 or later) and NEURON (7.0 or later, https://neuron.yale.edu/neuron/download) installed under Windows 7 or 10.
2. **'Simulations (NEURON)'** deals with NEURON-based simulations of cell biophysics and further adjustment of the astrocyte morphology (in accord with volumetric data); populating the cell with membrane mechanisms; setting up simulation configurations and protocols. This module can be run separately from other parts of ASTRO, it requires a Host computer with MATLAB (2013 or later) and NEURON (7.0 or later, https://neuron.yale.edu/neuron/download) installed under Windows 7 or 10. Please read the section **Preparing ASTRO system files** before attempting to run this module.
3. **'Ca^{2+} dynamics (Cluster)'** is to set up and simulate intracellular entry, diffusion, buffering, and extrusion of Ca^{2+} (and potentially other ions). Simulations of longer-term (seconds to minutes) intracellular calcium dynamics within realistic geometry with this module are likely to require the cluster / cloud-based parallel computing. This module can be run separately from other parts of ASTRO, it uses a Host computer with MATLAB (2012 or later) and NEURON (7.2 or later, https://neuron.yale.edu/neuron/download) installed under Windows 7 or 10, and, if required, a Worker computer / cluster operating under Linux and with preinstalled NEURON (https://neuron.yale.edu/neuron/download/compile_linux) and MPI (Message Passing Interface, see https://www.open-mpi.org/). Briefly, in the latter case the user working on the Host computer with MATLAB creates a MAT-file containing instructions for computation; uploads this file to the Worker cluster and launches there the simulations of astroglial Ca^{2+} dynamics (independently of the Host computer). The Host computer connects intermittently to the Worker cluster (a) to monitor computation progress, and (b) to download intermediate simulation results that are displayed and saved in MATLAB. Once simulations have been completed, the MATLAB module running on the Host computer downloads the output MAT-file and visualises the computation results.

   NOTE: The user can simulate Ca^{2+} dynamics with the Host computer only (no cluster), although the running speed could be low. The system has not yet been tested on MacOS or Linux which however support NEURON and Matlab.

Setting up and running ASTRO: Worker (cluster) computer preparation

Files from the GITHUB directory **Astro/clusterCaSim/hpc/** are to be uploaded to the cluster, with the folder structure kept unchanged (Fig. 4). The Worker computer should have NEURON (7.2 or later) installed beforehand.

To connect the Host and the Worker, the user should edit executable ***params.bat*** located on the Host computer:…**/host/Core/scripts/win-lin/*params.bat***

The program path defined inside the file ***params.bat*** has to be set to the cluster IP address (HEADNODEIP), the password of the cluster login (PASSWORD), the cluster login (LOGIN) and location of ***HPC*** file on the cluster (HEADNODEWORKERDIR).

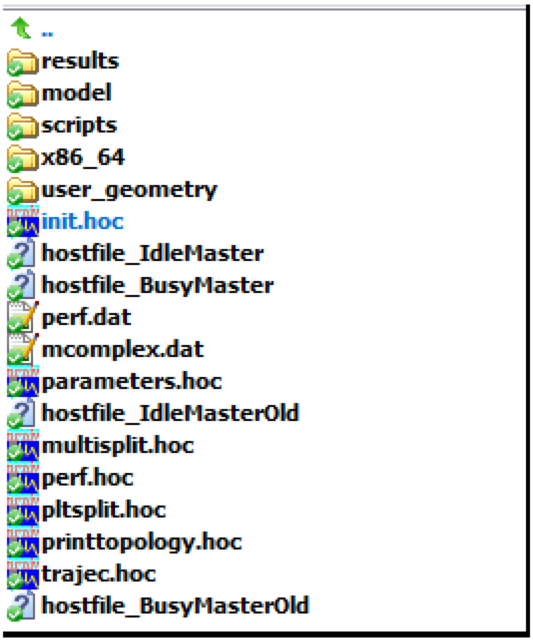

Figure 4. Folder structure for the Worker computer/cluster

Below is the corresponding fragment of the ***params.bat*** file.

```
****************************************
set HEADNODEIP=144.82.46.83
set LOGIN=my_login
set PASSWORD=my_password
set HEADNODEWORKERDIR=/home/*******/hpc
****************************************
```

ADOPTING EMPIRICAL DATA ON ASTROCYTE MORPHOLOGY

Main cell processes and branches

The first stage of morphological reconstruction deals with the main processes (stem tree) of the astrocyte under study. These are thought of as the processes that can be identified and traced in an optical (two-photon excitation) microscope. The procedure to digitise such structures in 3D and to save the data in a NEURON-compatible format is, in large part, similar to the well-established 3D reconstruction routines for nerve cells. The software (and detailed instructions) providing an output compatible with NEURON include, for instance, Vaa3D (Allen Institute) at

http://www.alleninstitute.org/what-we-do/brain-science/research/products-tools/vaa3d/

or NEUROLUCIDA (MBF Bioscience) at

http://mbfbioscience.com/neurolucida.

Alternatively, for exploration purposes the 3D stem tree can be downloaded from the database of nerve or neurogliaform cells NEUROMORPHO (http://neuromorpho.org/), or built independently using the function in NEURON function **'Create a cell'**.

Generating nanoscopic processes of astroglia

The MATLAB-based module **'Nano (Geometry)**' was designed with an aim: (a) to analyse the astrocyte nanostructure obtained using ultrathin serial-section 3D reconstruction, (b) to transform its 3D-reconstructed nanoscopic processes into NEURON-compatible shapes consisting of coaxial cylinders, and (c) to validate biophysical compatibility of the latter shapes with the original (irregular) 3D shapes.

The **'Nano (Geometry)**' module can either be activated from the introductory menu (Fig. 3) or launched separately from the folder **nanoGeometry** (Fig. 5):

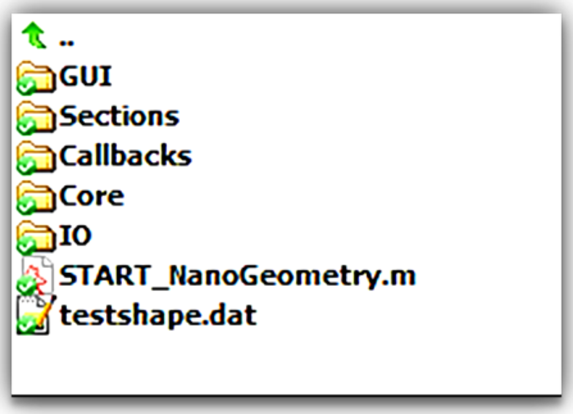

Figure 5. NanoGeometry folder containing the MATLAB code pertinent to the 'Nano (Geometry)' module of ASTRO.

Uploading a 3D-reconstructed fragment of astroglia

By default, the **nanoGeometry** folder (Fig. 5) hosts the ASCII-format ***testshape.dat*** file containing the XYZ surface coordinates (pseudo-random scatter, density 100-300 μm^{-3}) of an astrocyte fragment which was 3D-reconstructed using serial-section EM in the hippocampal area CA1 (*stratum radiatum*). The XYZ scatter includes points occurring along circumvents of the original serial sections. The reconstructed cell fragment contains multiple nanoscopic processes characteristic of the tissue-filling morphology of astroglia. .

These data provide the basis for obtaining key statistical properties of the nanoscopic shapes to be transformed into NEURON-compatible, cylinder-compartment-based nanoscopic astroglial processes. Whilst the fine architecture of astrocytes in CA1 neuropil could be characteristic of various brain regions, the user is also encouraged to generate their own ***testshape.dat*** file containing the 3D-EM data (micron units) representing the astroglia of interest. The method of obtaining the surface point scatter in the 3D reconstruction data could vary depending on the software involved.

Storing a representative sample of 3D-reconstructed nanoscopic processes

With ***testshape.dat*** file in place, the **'Nano (Geometry)'** module can be launched from the introductory menu (Fig. 3) or by executing the file ***START_NanoGeometry.m***. This prompts two adjacent windows (Fig. 6) which display the digital data from the ***testshape.dat*** file (Fig. 6a) and a list of shapes (Fig. 6b).

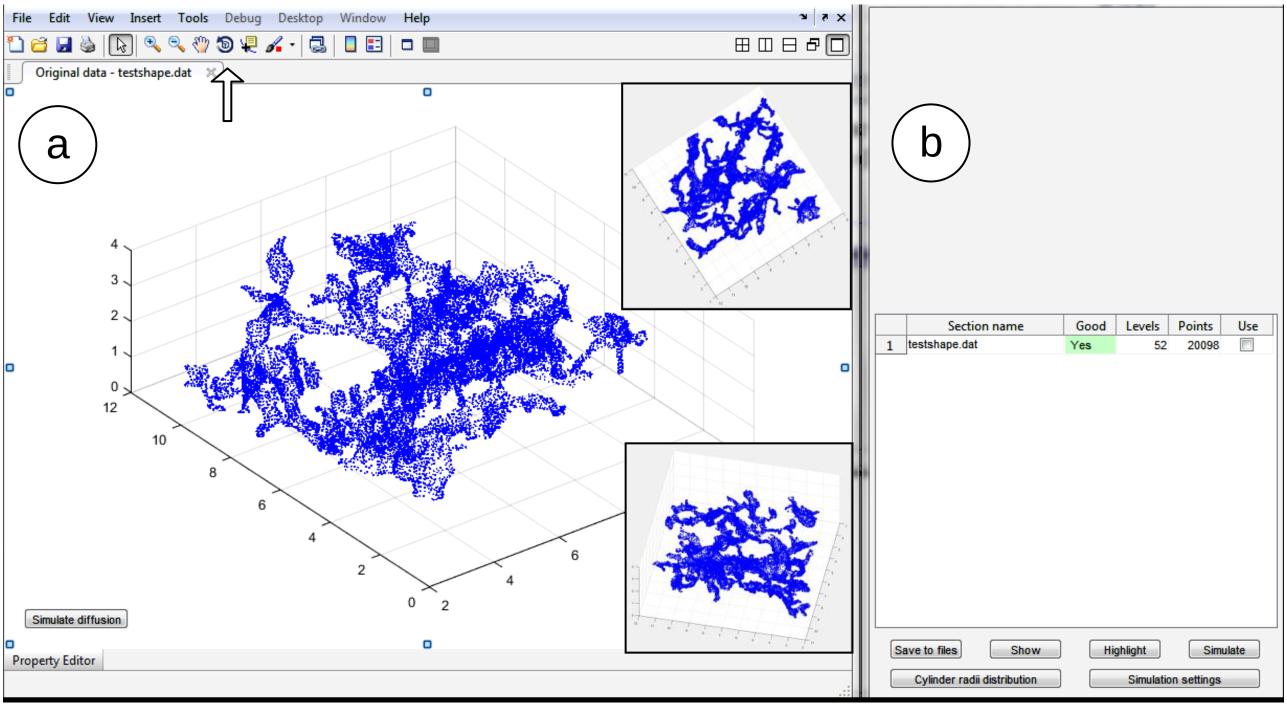

Figure 6. Window panels to visualise and select nanoscopic astroglial processes extracted from the 3D data file *testshape.dat*. a, 3D presentation of the structure (surface point scatter delineating serial sections); arrow points to the menu control knob for 3D shape rotation; two insets (not to appear on the interface monitor) display example snapshots of the same structure rotated in 3D. **b,** A list of the selected 3D-reconstructed shapes (one shown, to correspond to one imported file).

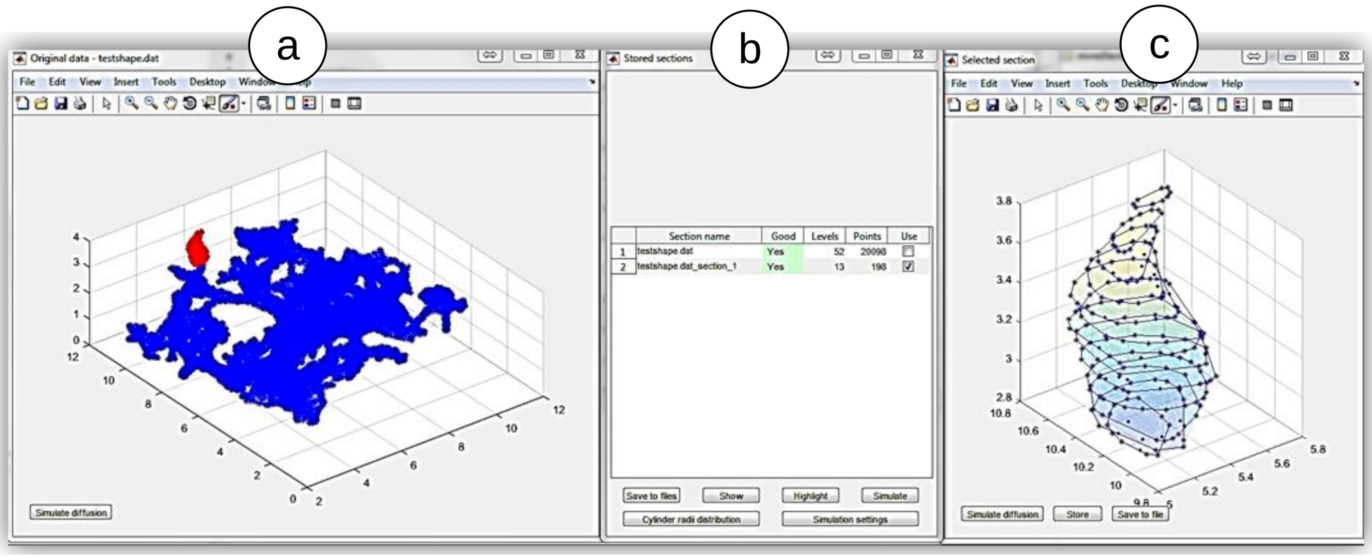

Figure 7. Window panels: selection and analysis of 3D-nanoscopic processes. a-b (as in Fig. 6)**,** one process is selected with the 'Brush' tool (highlighted in red). **c,** the selected process (shown in red in **a**) magnified and shown as a set of serial sections outlined as polygonal shapes by data points (original data) that represent the rendered surface of the structure.

Here, the aim is to collect a representative sample of 3D-reconstructed nanoscopic processes, which would accumulate the morphometric statistics suitable for generating NEURON-compatible shapes based on cylindrical compartments. The 3D structure could be rotated (Fig. 6a) to view and select individual processes using the 'Brush' selection tool, which 'paints' sampled structures in red (Fig. 7a).

The selected process (highlighted in red) is immediately displayed, with the corresponding surface points (original data) representing polygonal serial sections (Fig. 7c). In this display window (Fig. 7c), the user can examine the process shape using the functions shown on the menu (window top). The unwanted (erroneously sampled) data points, such as those from neighbouring processes, could be deleted using 'delete/remove' features of the **'Brush'** menu (mouse right key).

Once the user has been satisfied with the selected process, its structure can be added to the statistics accumulation list using **'Store'** menu option (Fig. 7c). The selected structure should be suitable for statistics accumulation, in which case it is displayed (Fig. 7b) as **'Yes'** and shown in green. Some shapes could appear mathematically unusable (due to data outliers, topological confusion, or insufficient data point numbers), in which case they will be indicated as **'No'** and shown in red.

Fig. 8 panel illustrates the **'Stored selections'** window (as in Fig. 7b) containing five processes. To accumulate sufficient shape statistics, it is recommended to select and store at least 15-20 individual processes for further analyses.

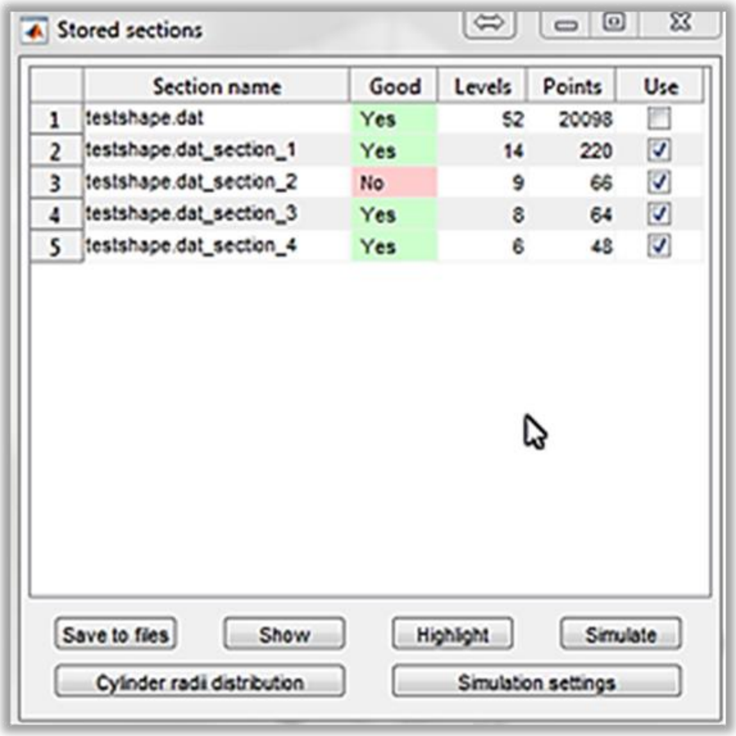

Figure 8. Window panel displaying the morphology sample file name (*testshape.dat*) and file names of four selected processes (shapes not shown). Process No 3 (shape not shown) appears unsuitable for analyses: it is excluded by unclicking.

The menu in the **'Stored selections'** window (Fig. 8) has six executive options, as briefly outlined below (and explained in further details in subsequent sections):

'Save to files': Data will be saved to a file;

'Show': The selected processes will be shown in details (as in Fig. 7c);

'Highlight': The selected processes will be highlighted on the sampled structure (as in Fig. 7a);

'Simulate': This menu option will launch Monte Carlo simulations (Brownian motion of neutral molecules or charged ions through the selected process) testing the biophysical features of the selected process;

'Cylinder radii distribution': Calculating statistical data for the selected structures;

'Simulation settings': Setting parameters of Monte Carlo simulations.

The ultimate aim at this stage of astrocyte model building is to generate the cylinder diameter statistics that would represent real (3D-reconstructed) processes using sets of NEURON-compatible cylindrical (disk-shaped) cellular compartments, as explained in the sections below.

Transforming 3D-reconstructed processes into sets of disk-shaped compartments

Once the user has selected one nanoscopic process for analyses (Fig. 9a), it will be displayed as a 3D structure consisting of *N* serial sections formed by *N* polygon-based slabs (Fig. 9b). The number of slabs *N* making up the shape normally matches the number of ultrathin (EM) serial sections in the original structure.

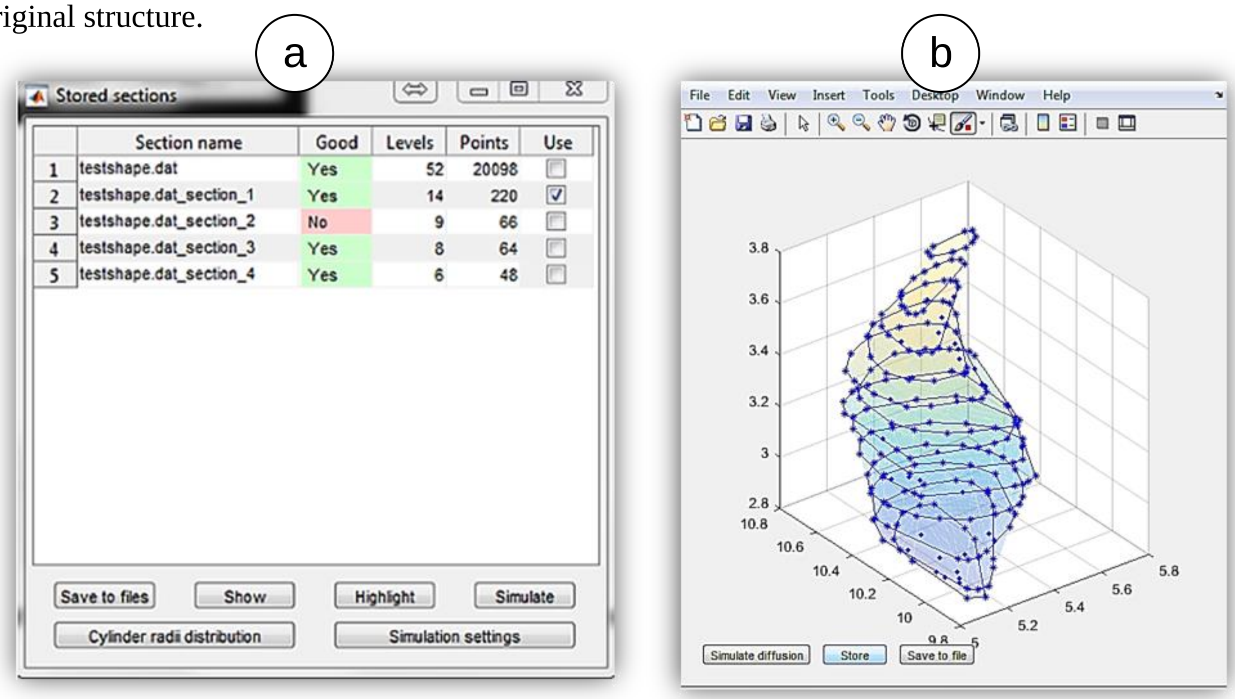

Figure 9. Window panels to explore the geometry of selected and stored processes. a, Panel (as in Fig. 7b) showing one process selected for analyses. **b,** The selected process displayed in detail as a set of layers represented by polygonal slabs (as in Fig. 6c)**.**

Here, the task of the '**Nano (Geometry)'** module is to transform these irregular polygon-based structures into coaxial circular cylinder-based structures with similar geometrical (and biophysical) properties. To achieve this, each pair of the adjacent irregular polygonal slabs, which are expected to be joined eccentrically, is transformed into three disk-shaped slabs (round cylinders), two 'regular' and one 'transitional' (Fig. 10a). The base area of a regular cylinder corresponds to that of the polygonal slabs (Fig. 10a, blue) whereas the base area of the transitional cylinder is the interface area between adjacent polygonal slabs (green segments in Fig. 10a). Thus, the cross-section and side area properties of the original structures are, in large part, preserved in the cylinder-based structure.

By repeating this routine, '**Nano (Geometry)'** transforms the original polygon-based shape (Fig. 10b) into a cylindrical structure consisting of 2*N*-1 cylindrical slabs (*N* regular and *N*-1 transitional; Fig. 10c). It then calculates the distribution of the cylindrical slab diameters.

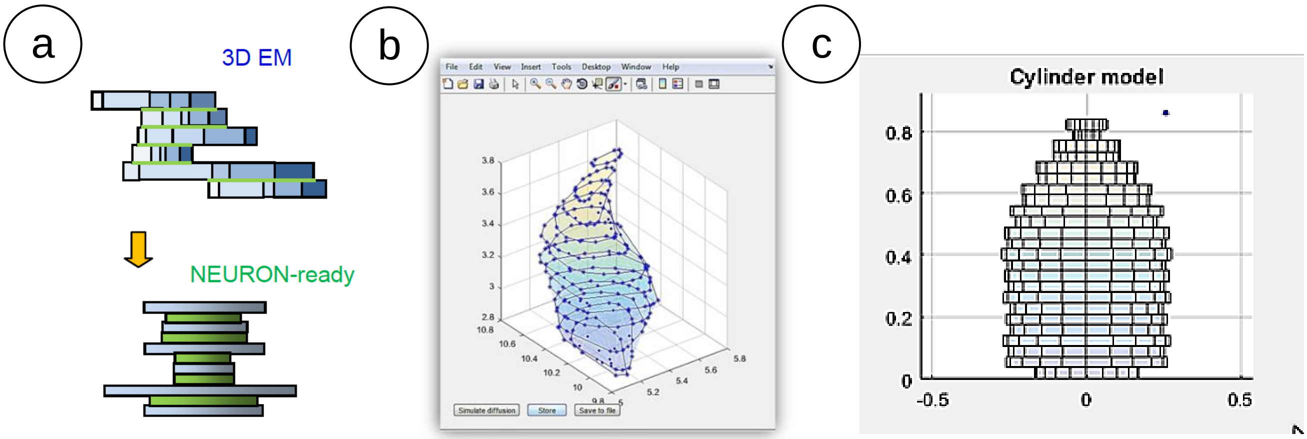

Figure 10. Transformation of irregular polygon-based structures into coaxial cylinder-based structures. a, Diagram illustrating the transition from an irregular z-stack of polygonal slabs (top, blue; green segments indicates contact surface) into a stack of coaxial cylindrical slabs (bottom; transitional slabs shown in green). The base area of the transitional cylinders (bottom, green) matches the slab-joint areas (top, green segments). **b,** The original structure (as in Fig. 9b) consisting of *N* polygonal slabs. **c,** Structure in **b** transformed into a NEURON-compatible structure consisting of 2*N*-1 coaxial cylinders (disks).

Biophysical comparison of original and NEURON-compatible structures: Monte Carlo test settings

The key test for physical compatibility of the original (polygon-based) and transformed (cylinder-based) structure is the dynamics of particle flux through the structure. The **'Nano (Geometry)'** module is equipped with a Monte Carlo experiment generating Brownian diffusion and electrodiffusion (i.e., electric current) of free particles injected instantaneously at one end of the structure (Z=0) and monitored for their escape at the other end. Simulations are displayed in 'real-time' producing two plots of the particle flux dynamics for both structure types. Before running the test, the user should set up its parameters using **'Simulation settings'** (menu choice in Fig. 9a) and then **'Sim parameters'** options (Fig. 11), as outlined below.

In this window (Fig. 11a), the options are as follows (notations are given in accord with MATLAB variable names). **'simComplexity'** provides the choice between **Simple** (faster but less precise) and **Complex** (slower but more precise) modes of simulation. The **Simple** mode employs a straightforward Monte Carlo algorithm for the interaction of particles with the astrocyte plasma membrane: the stochastic trajectory of diffusing particles is not permitted to go beyond the structure boundary (cell wall). In contrast, the **Complex** mode explicitly calculates elastic-collision interactions of individual diffusing particles with cell walls.

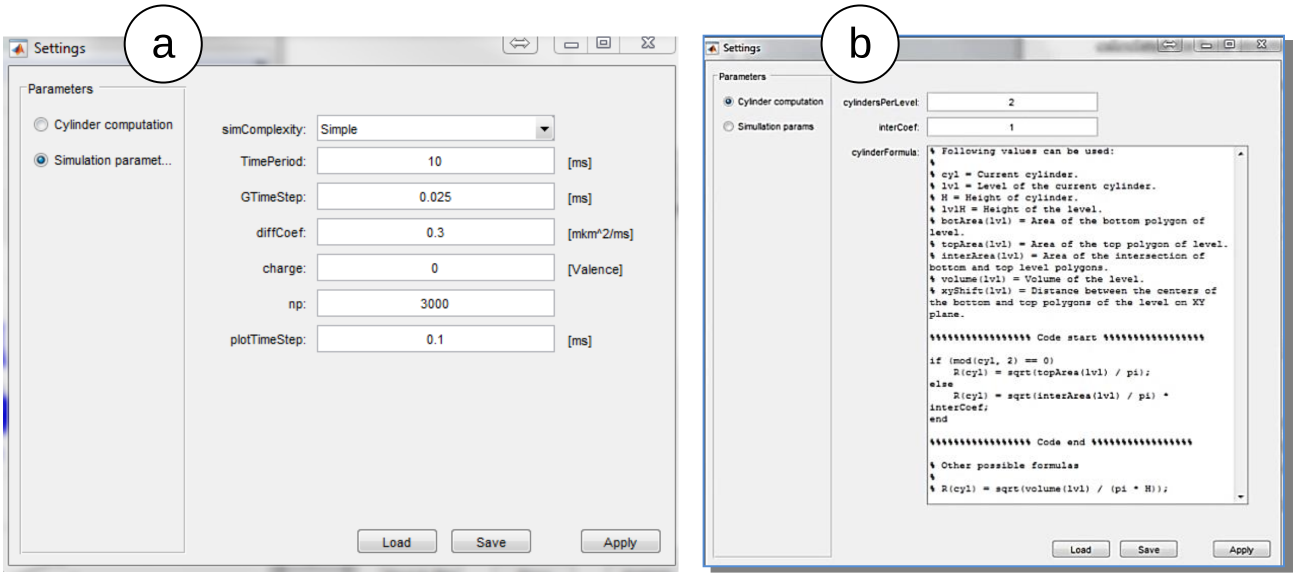

Figure 11: Window panels to set Monte Carlo simulations of Brownian diffusion and electric current (electrodiffusion) flow through the selected nanoscopic structure. Default settings and parameter values are shown.

'RunTime', simulation run time (ms);
'GTimeStep', diffusion simulation time step (ms) ;
'DiffCoef', diffusion coefficient (μm^2/ms);
'Charge', particle charge (0 for free diffusion, 1 for electric current);
'Np', total number of simulated particles;
'plotTimeStep', time step in the outcome data plot.

The electric field by definition is $2.5 \cdot 10^3$ V/m. The change of the electrofusion is possible by changing the original particle charge.

The **Settings** panel (Fig. 11b) is designed primarily for algorithm error checking, in-depth analyses, and further software development: it displays the MATLAB code for cylinder-shape parameters. Parameter **'CylinderPerLevel'** sets the number of cylinder slabs per polygonal slab.

Biophysical comparison of original and NEURON-compatible structures: running the test

Pressing **'Simulate diffusion'** key (Fig. 11a) opens window panels displaying Monte Carlo simulation progress (Fig. 12a) and its outcome (Fig. 12b-c).

When the particle flow dynamics are indistinguishable between the original and cylinder-based nanoscopic processes (Fig. 12b-c), this indicates that the diffusion properties of the two structures are similar. A satisfactory match implies that the transformed (NEURON-compatible, cylinder-based) structure can be added to the statistics of cylinder diameters used in the NEURON script to generate astroglial processes. The latter is done using '**Save to file**' option (Fig. 9b).

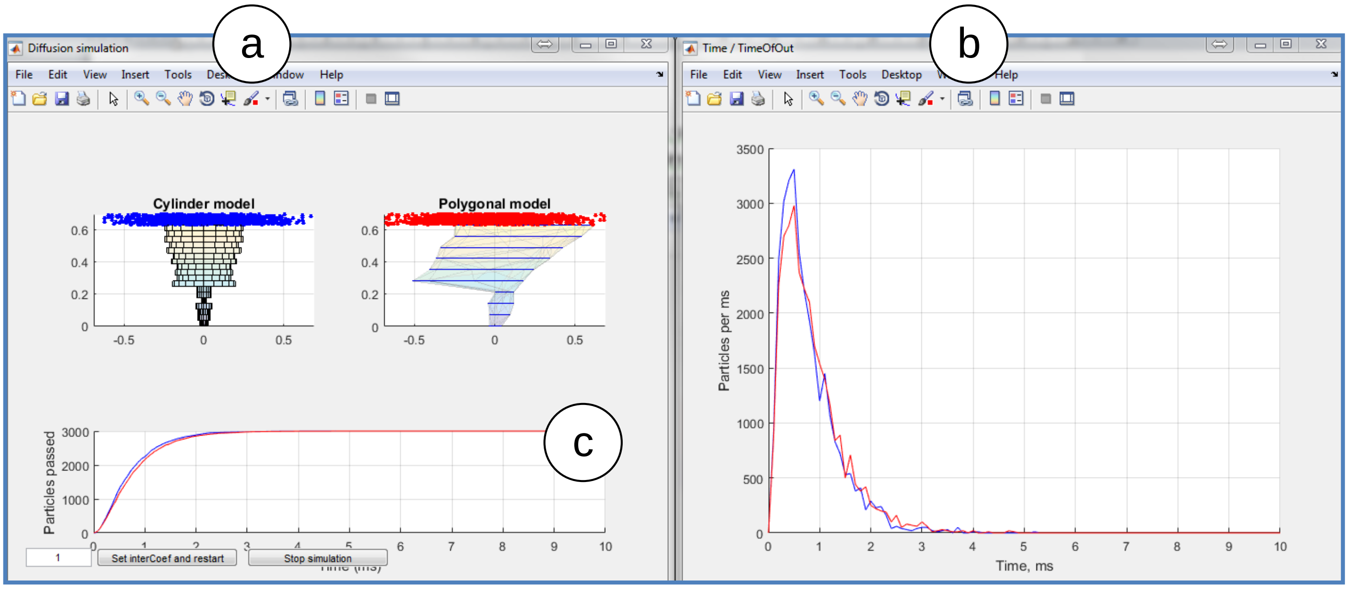

Figure 12. Monte Carlo simulations of particle flux dynamics in the original and the cylinder-based nanoscopic processes. **a**, Two structures shown, cylinder-based transformed process (left) and the original polygon-based process (right). **b**, summary of the particle flux (number of particles per milliseconds) escaping the cylinder-based process (blue) and the polygon-based process (red). **c**, Monitoring the particle escape (particles passing through the structure).

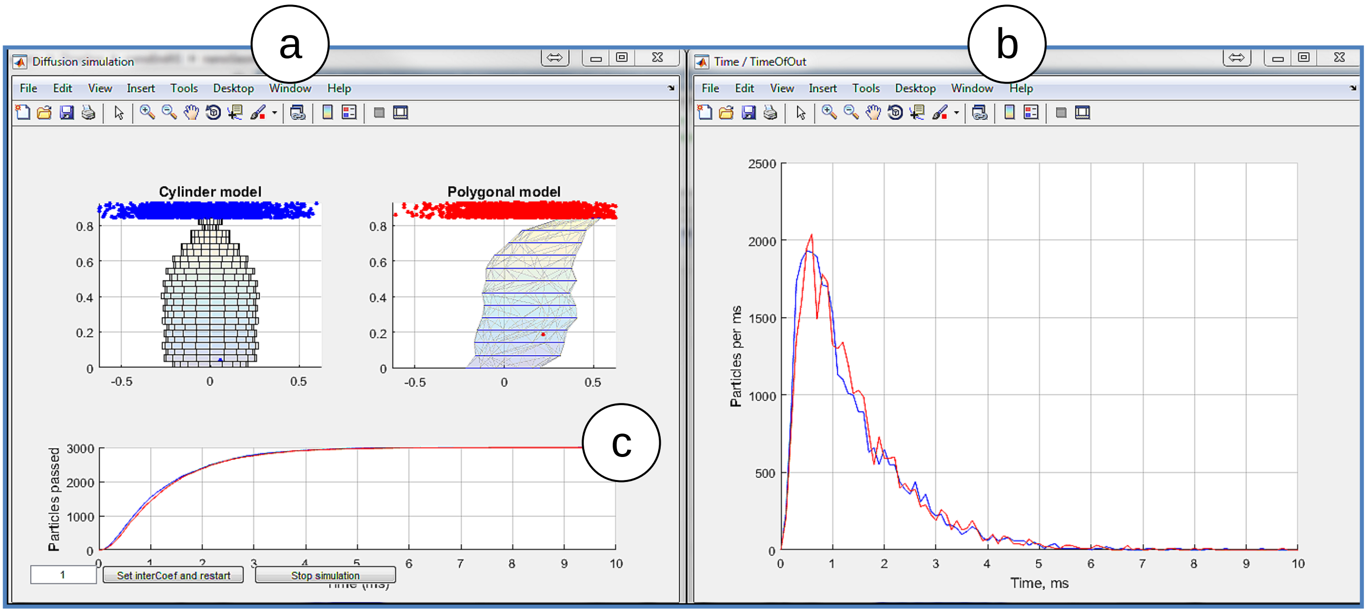

Figure 13. Example of Monte Carlo simulations (as in Fig. 12) indicating that the cylinder-based structure (a, left) lets through Brownian particles at a higher rate than does the original polygon-based structure (b-c). This requires an adjustment of transitional cylinder diameters using **'Set interCoef'** button (a bottom left). Other notations as in Fig. 12.

A similar procedure will test and compare the electric current (electrodiffusion) properties between the two structures, by setting non-zero **'Charge'** (Fig. 11a): the default field strength is $2.5 \cdot 10^3$ V/m, which corresponds to a **'Charge'** value of 1; other **'Charge'** values will adjust the electric field strength proportionally.

When the molecular flux dynamics are somewhat dissimilar (example in Fig. 13), the user can adjust effective diameters of transitional cylinders using the **'Set interCoef'** option (Fig. 13c): reducing the average transitional diameter will slow down the particle fluxes, and *vice versa*. Following the adjustments, simulation should be repeated until the readout of particle dynamics are matched between the structures (Fig. 14). The adjusted structure is thereafter added to the data pool generating the statistics of cylinder diameters.

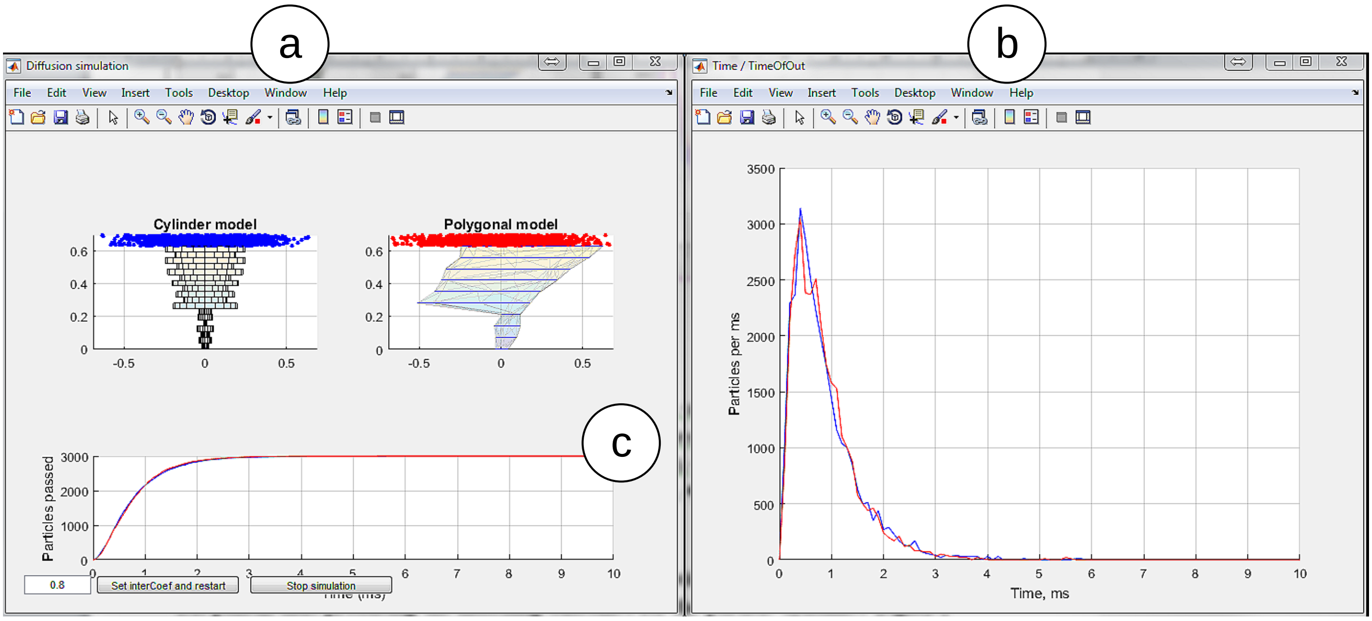

Figure 14. Monte Carlo simulations, as in Fig. 13, but with the adjusted transitional cylinders (a, left) thus producing the matching outcomes for the particle dynamics (b-c). Notations as in Fig. 13.

Statistics of NEURON-compatible cylindrical compartments

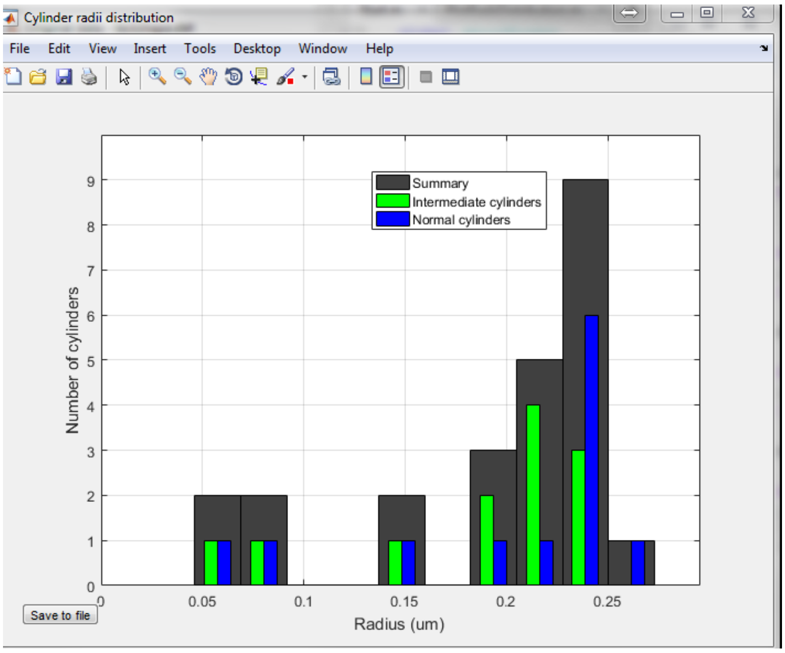

Once the accumulation of data on individual cylinder-based structures is complete, pressing **'Cylinder radii distribution'** (Fig. 8 panel) generates the distribution of cylinder-compartment diameters representing the astroglial processes (Fig. 15; one process data are shown for the sake of clarity).

Figure 15. Distribution of cylinder-compartment diameters: data based on Fig. 10b example of a single nanoscopic process. Blue and green, regular and transitional cylinders, respectively (Fig. 10a); black, cylinders combined.

This resulting distribution (from multiple nanoscopic processes) can be saved into a data file using the **'Save to files'** button (Fig. 8). This distribution will be used to generate nanoscopic astrocytic processes with the NEURON script, as explained in the sections below.

GENERATING COMPLETE ASTROCYTE MORPHOLOGY

File structure in Host computer (under Windows)

This section explains initial steps to launch and run the NEURON environment adapted for astroglial modelling, on the Host computer under Windows.

Preparing ASTRO system files

1. Set the path to NEURON on the Host computer using the batch file ***neuron_home_win.bat***; by default, it is set as *NEURON_HOME_WIN='C:\nrn'*.
2. Execute the ***init.hoc*** file located in the host computer directory *…neuronSims\init.hoc*' or use the button 'NEURON simulations' from the start menu panel (Figs. 2-3).
3. Activate the main panel (Introductory menu; Figs. 2-3), by executing ***Start.m*** (and then Run button) in MATLAB. Here ASTRO triggers compilation of the NEURON *.mod files automatically. Alternatively, create the NEURON executive mod-files by running ***mknrndll.exe*** (NEURON installation directory) and afterwards setting the working directory of ASTRO *...\neuronSims*

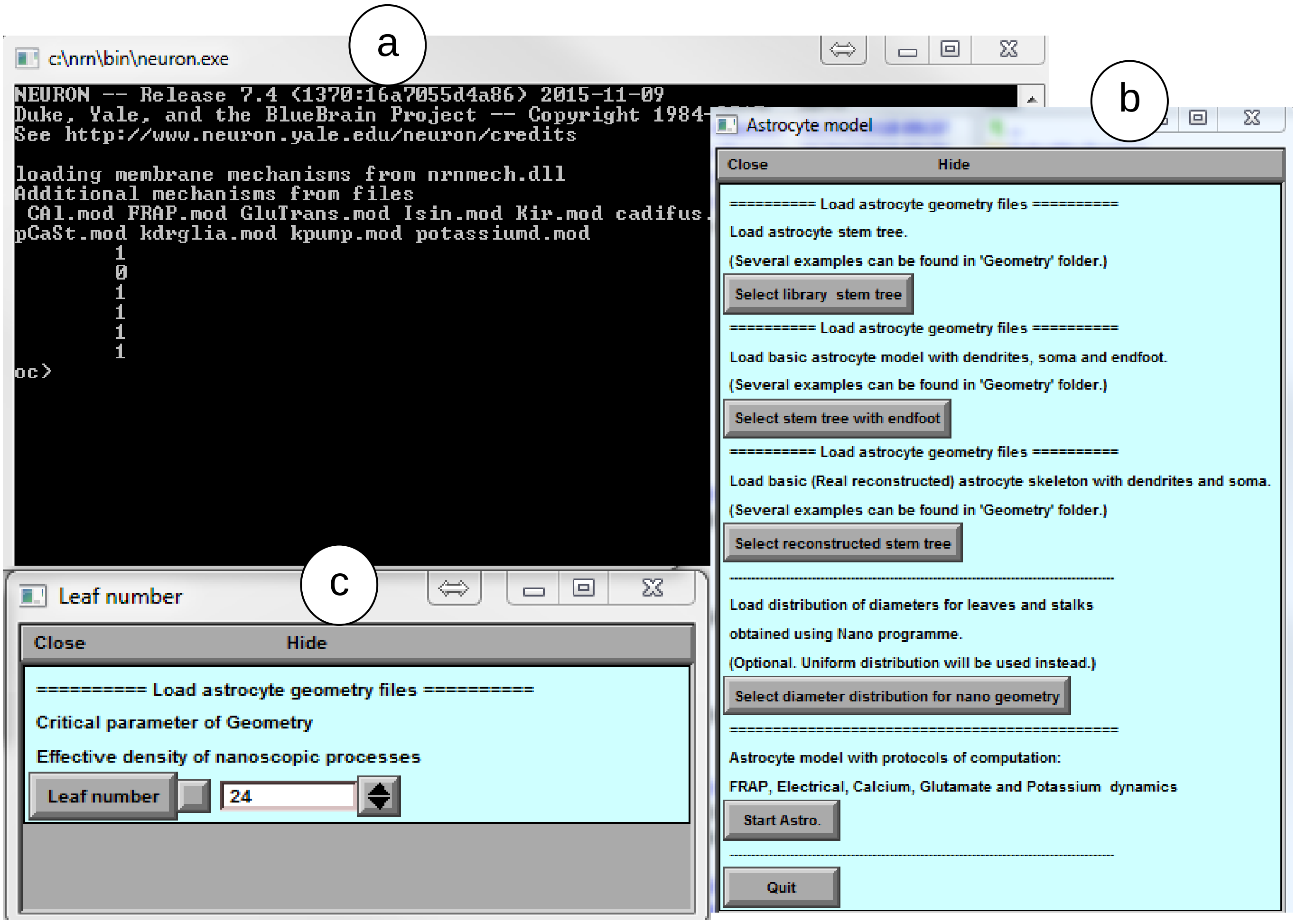

Figure 16. Control windows initiated by launching NEURON in the ASTRO environment, as detailed in the text.

4. The file ***init.hoc*** opens three windows (Fig. 16): System window (***cmd.exe***) (Fig. 16a), a window panel to define the gross astrocyte geometry (stem tree, Fig. 16b), and a menu panel to set the density for higher orders of nanoscopic processes ('**Leaf number**'; Fig. 16c).

Generating astrocyte stem tree (including endfoot)

To design a new astrocyte model, the user has to define the basic structure of dendritic tree using three different options:

Option 1: **'Select library stem tree'.** This option is to import hoc-files from the database http://neuromorpho.com or from the in-house directory. Some example files are provided in the directory .../*Geometry* (Fig. 17a); they can be used as a file format guide. For the geometry file to connect to ASTRO, it should have the following initial script:

```
**************************************************************************
OriginalDendrite=133 // This number should correspond to the number of main branches of the
//3D reconstructed geometry
NumberDendrites=OriginalDendrite+2*(OriginalDendrite-1)
SeedNumber=OriginalDendrite-1
create soma[1]
create dendrite[NumberDendrites]
access soma[0]
**************************************************************************
```

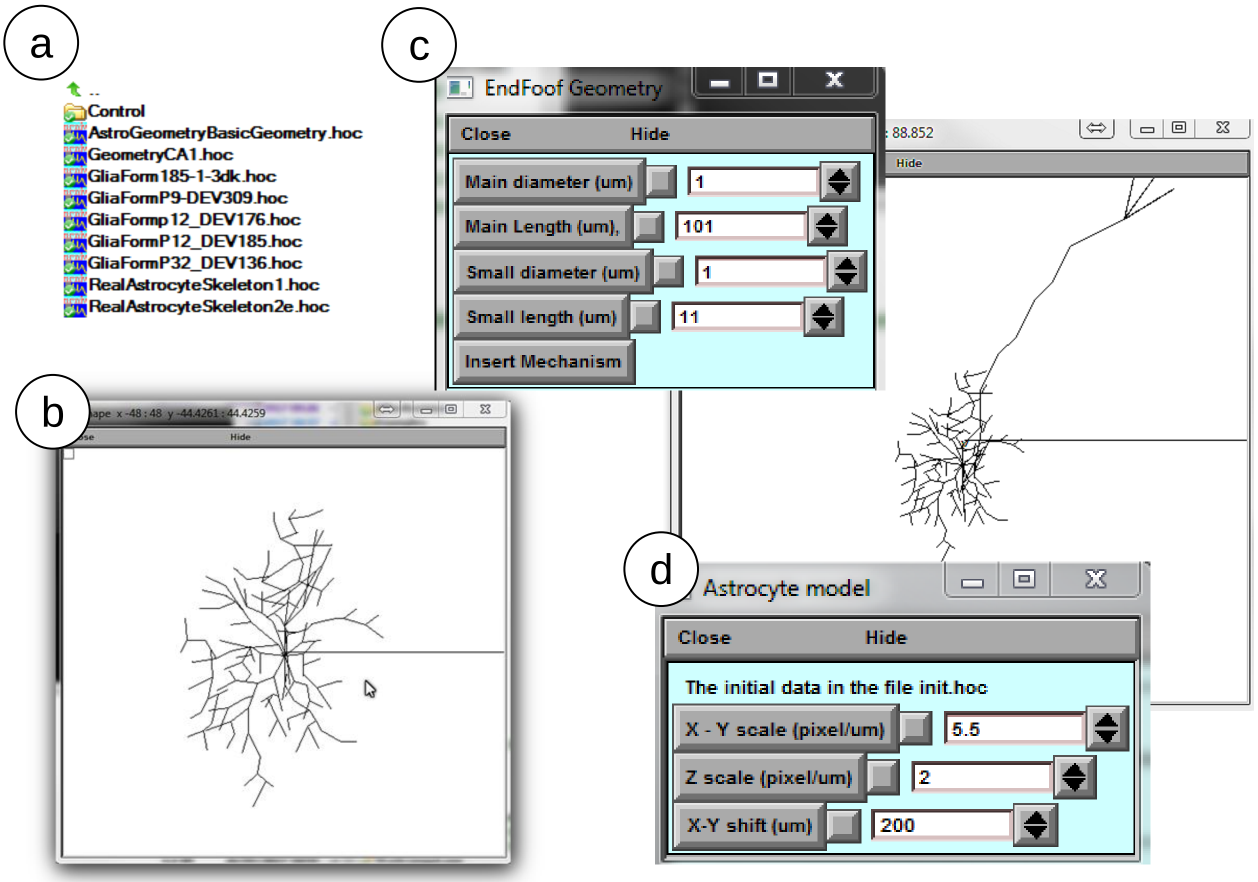

Figure 17. Operational window panels pertinent to the creation of gross astroglial morphology including the endfoot feature.

where OriginalDendrite sets the number of branches (dendrites in NEURON terminology) on the stem tree. The database ***NeuroMorpho.org*** can be used as a guide to the ASTRO-compatible file format. Upon selection, a window panel is activated displaying the selected stem tree (Fig. 17b).

Option 2: 'Select stem tree with endfoot' is similar to Option 1, but with the endfoot structure, which is stochastically generated (Fig. 17c). Here, an additional window panel is activated (Fig. 17c, left), providing a menu to set the morphology of the main and the secondary endfoot branches, and to set up the local biophysical mechanisms.

Option 3: 'Select reconstructed stem tree' loads the 3D-reconstructed stem tree file. An example in ***RealAstrocyteSkeleton1.hoc*** (the directory *…/Geometry*) shows the reconstructed stem tree of the CA1 astrocyte using the Vaa3D software (Allen Institute, available from http://www.alleninstitute.org/what-we-do/brain-science/research/products-tools/vaa3d/). This option also prompts an additional window panel (Fig. 17d), providing setting for geometrical scaling and the centring of the astrocyte structure at the coordinate origin (to facilitate positioning of selected cell compartments). The corresponding menu buttons thus include '**X-Y scale (pixel/μm)**', '**Z scale (pixel μm)**', and '**X-Y shift (μm)**'. This window will disappear after any parameter change.

NOTE: The user must upload the cell stem tree geometry before initiating any further design of the model.

Generating astroglial morphology on the nanoscale

Geometry of nanoscopic processes

Once the stem tree has been downloaded, the next stage is the nanostructure of astrocyte. The user has two options.

Option 1: To download the default nanostructure prepared in advance. Pressing the button **'Diameter distribution for nano-geometry'** (Fig. 16b) prompts the use to download a file with the statistics of process diameters produced by the '**Nano (Geometry)'** module from the sampled 3D-reconstructed astroglial processes (see above). By default, this option downloads the file ***testshape.dat_radii_dist.txt*** (characterising astroglial processes in CA1 *stratum radiatum*). After that, the user presses the '**Start Astro'** button (Fig. 16b).

Option 2: To press '**Start Astro'** button, in which case ASTRO generates nanoscopic processes automatically using the built-in tools.

In both cases, the user can repeatedly adjust key morphometric features of the generated nanostructures. See further details in the chapter **Simulating Astrocyte Physiology**.

Populating astrocyte tree with nanoscopic processes

The '**Start Astro**' button (Fig. 16b) prompts the main window panel '**Repertoire of computation**' (Fig. 18), which is key to the modelling of complete astrocyte morphology, as described in the sections below.

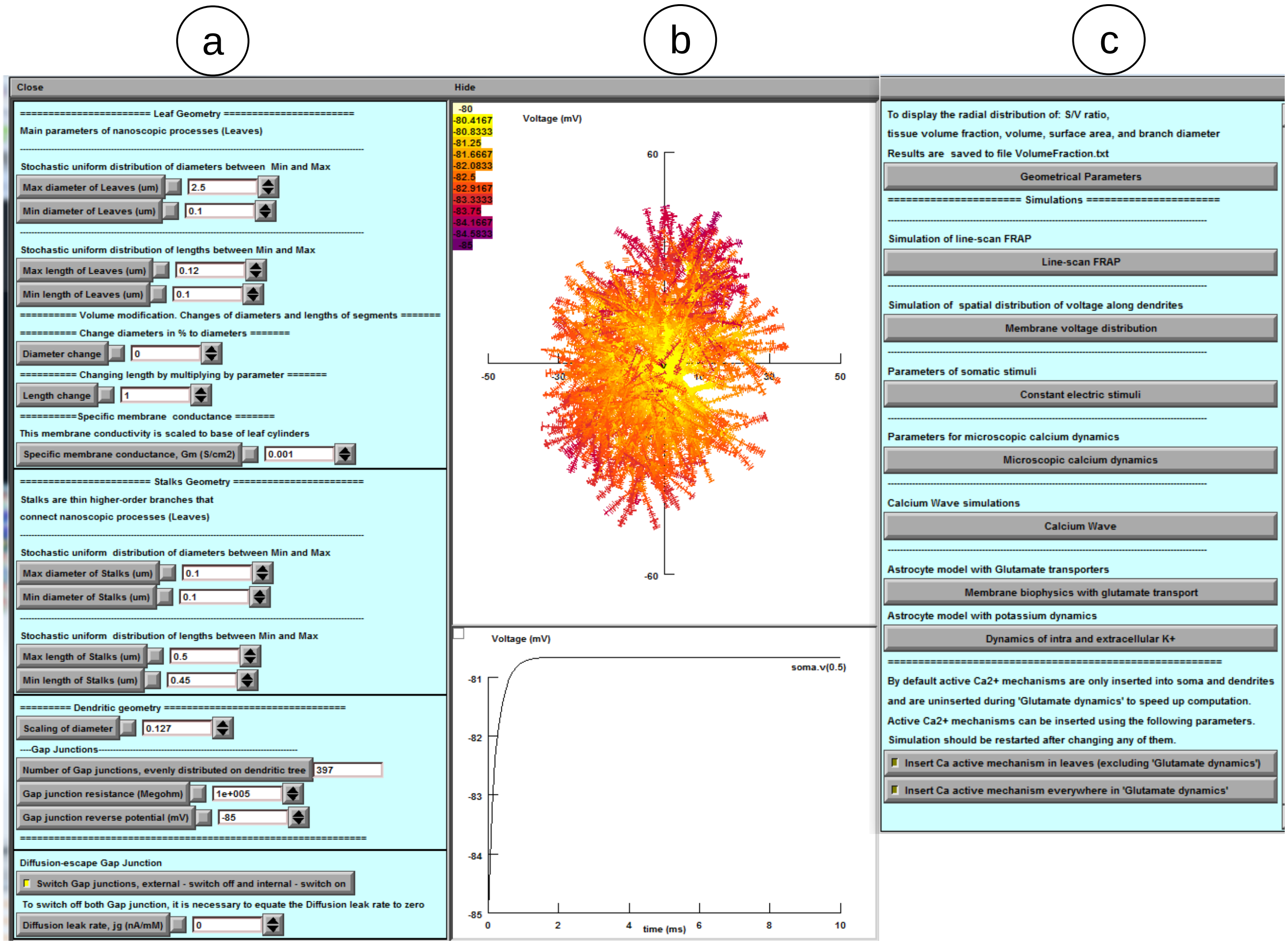

Figure 18. Main window of ASTRO. a, Control panel providing detailed settings of astrocyte geometry including a gap junction feature (bottom). **b,** Simulated variable mapped onto astrocyte morphology (top; membrane voltage shown), with selected digital output plot (bottom). **c,** Computational scenarios with parameter setting.

Panel '**Leaf Geometry**' (Fig. 18a, top) provides an option to set up the distribution of cylindrical compartments (leaves) of nanoscopic processes as evenly random (with lower and upper limits) when the experimental statistics on 3D reconstructed processes are not available.

NOTE: This section is to be ignored when the latter have already been loaded (see previous section).

Panel '**Stalk Geometry**' (Fig. 18a, middle) sets upper and lower limits for the uniform distribution of transitional cylinders of nano geometry. These parameters determine how densely the tissue is to be filled with nanoscopic astroglial processes.

Panel '**Specific membrane conductance**' sets this value at the button '**G_m (mS/cm^2)**', which takes into account all exposed surfaces of the cylindrical compartments. Resting potential of the current is -85 mV. This parameter is defined on the built-in NEURON panel “Distributed mechanism”.

Panel **'Dendritic Geometry'** (Fig. 18a, bottom) currently includes **'Branch diameter scaling',** which sets the scaling coefficient for the stem tree branch diameters as a function of distance from the soma, according to the average experimental trend. The empirically established formula for the branch diameter *d* is $d \sim (S(r+1))^{-1/2}$ where **'scalingDiam'** value S and *r* is the distance to the soma.

NOTE: This panel has to be ignored if a 3D-reconstructed stem tree has been uploaded.

Panel **'Gap junctions'** is explained in the **Gap Junctions** section below (chapter **Simulating Astroglial Function**).

Tissue-filling properties of astroglial morphology

The tissue volume-filling properties and the surface-to-volume ratios of the nanoscopic processes will be determined by the shapes and the effective density of simulated nanoscopic processes, as described in the previous section. Tissue volume filling and other geometry features of the model can be monitored by pressing **'Geometrical parameters'** key (Fig. 18c, top): this opens several window panels displaying various parameters of the modelled cell geometry (Fig. 19). The displayed data are automatically saved to the file ***…\neuronSims\Text results\VolumFraction.txt***.

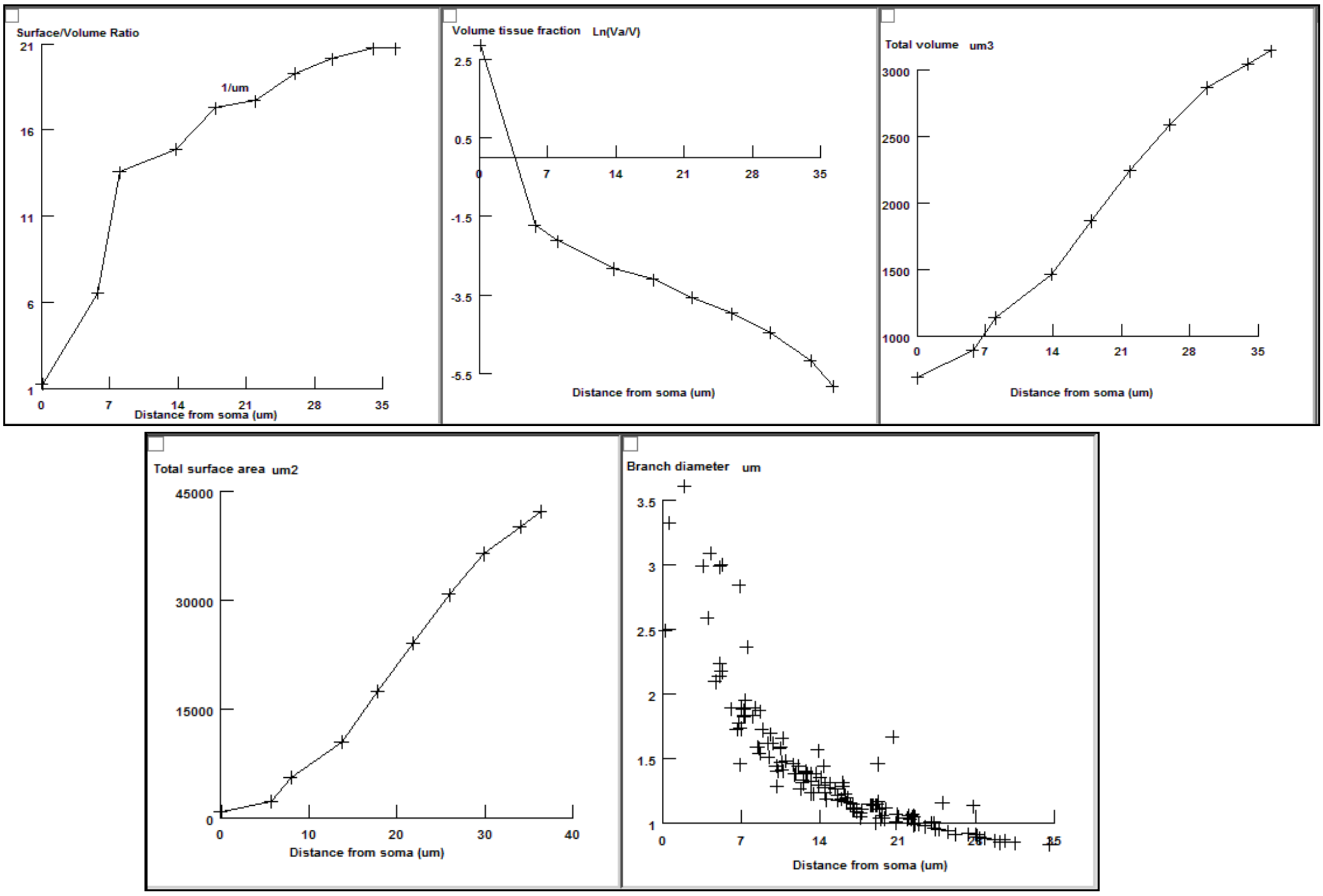

Figure 19. Window panels providing readout of the volumetric characteristics for modelled astroglia (launched by 'Geometrical parameters' button). From top left: surface-to-volume ratio distribution, tissue volume fraction, total cell volume (cumulative value with the distance from the soma), total cell surface area, diameters of main processes.

The morphometric characteristics of the simulated astrocyte (Fig. 19) are to be compared with the corresponding empirical data obtained using 3D EM reconstructions and two-photon excitation

imaging data for the astroglia of interest (Fig. 1). The user is free to evaluate the mismatch and adjust the density of nanoscopic processes (using '**Stalk Geometry**' and '**Dendritic Geometry**' options where relevant; Fig. 18) correspondingly, until an acceptable match is produced. The windows depicting critical geometrical parameters (Fig. 19) can be viewed at any time during modelling.

At the end of this stage, the modelled astroglial morphology is complete (see 'FRAP experiments' below for further subtle morphological adjustments). The user can begin to simulate various functions of astroglia while also implementing a variety of membrane and intracellular biophysical mechanisms, as briefly explained in the sections below.

Generating and exploring pre-determined astrocyte models

There are currently two pre-set models of the astroglia stem tree (main processes): one is obtained from a 3D reconstructed cell, stored in file ***GeometryAstrocyteCA1.hoc***, and the other is a 'typical' CA1 astrocyte (i.e. the cell whose macroscopic and nanoscopic features represent average values over a sample of CA1 astrocytes), stored in file ***AstrocyteBasicGeometry.hoc***. The pre-set nanoscopic geometry representing the main features of nanoscopic astroglial processes in area CA1 (obtained from 3D EM reconstructions) is stored in file ***testshape.dat_radii_dist.txt***.

To download and work with one of the pre-set astrocyte models:

1. In the main NEURON menu (Fig. 16), set **'Leaf number'** option (Fig. 16c) up to 50.
2. Press **'Select basic geometry uploaded...'** menu button (Fig. 16b).
3. In the pop-up menu go to the .../*Geometry* and select ***GeometryAstrocyteCA1.hoc*** for a 'real CA1 astrocyte' geometry or ***AstrocyteBasicGeometry.hoc*** for 'typical CA1 astrocyte' geometry.
4. Press **'Select diameter distribution for the Nano geometry'** menu button (Fig. 16b).
5. In the pop-up Load menu select ***testshape.dat_radii_dist.txt*** file**.**
6. Press **'Start program'** (Fig. 16b).

The program will prompt the main control panel (**'Repertoire of computation'**, Fig. 18) displaying the newly-generated multi-scale model of an astrocyte (Fig. 18b).

Subsequent versions of ASTRO will include additional pre-set models of the astrocyte tree stem and its nanoscopic geometries.

SIMULATING ASTROGLIAL FUNCTION

Linescan FRAP experiment: probing intracellular connectivity of astroglia

Fluorescence recovery after photobleaching (FRAP) applied with respect to water-soluble intracellular indicators assesses effective diffusivity across the cellular compartments adjacent to the bleached area. FRAP experiments and the corresponding simulations (launched from the main ASTRO window; Fig. 18c) could be used therefore to test whether the modelled astrocyte morphology reproduces intracellular diffusivity (connectivity) properties documented empirically. The default values correspond to the data obtained in CA1 astroglia using linear (**linescan**) photobleaching tests with Alexa Fluor 594 or Alexa Fluor 488.

This test simulates FRAP with a photobleaching area represented by a segment (**linescan**) in the XY plane. Pressing **'Linescan FRAP'** opens its control window (Fig. 21):

The initiation panel (Fig. 21a) provides the following settings:

'Linescan width' sets the linescan width: in two-photon excitation mode, the point-spread function of the focused laser beam is on average 1-1.5 µm depending on the system optics; this parameter is therefore adjustable (within a narrow range).

'Angle' and **'Y at x=0'** set, respectively, the inclination angle and the y-axis intersect of the photobleaching (scanning) segment.

'Initial concentration (mM)' sets the basal concentration of the photo-bleachable dye molecules, to match the experimental value (in configuration of whole-cell dialysis).

'Photobleaching rate' sets the rate of photobleaching (free parameter that can be used to match simulated and experimental data).

'Dfree' sets the intracellular diffusivity of the dye molecules based on empirical estimates.

'Bleaching recovery interactions' sets the number of bleaching-recovery periods per trial.

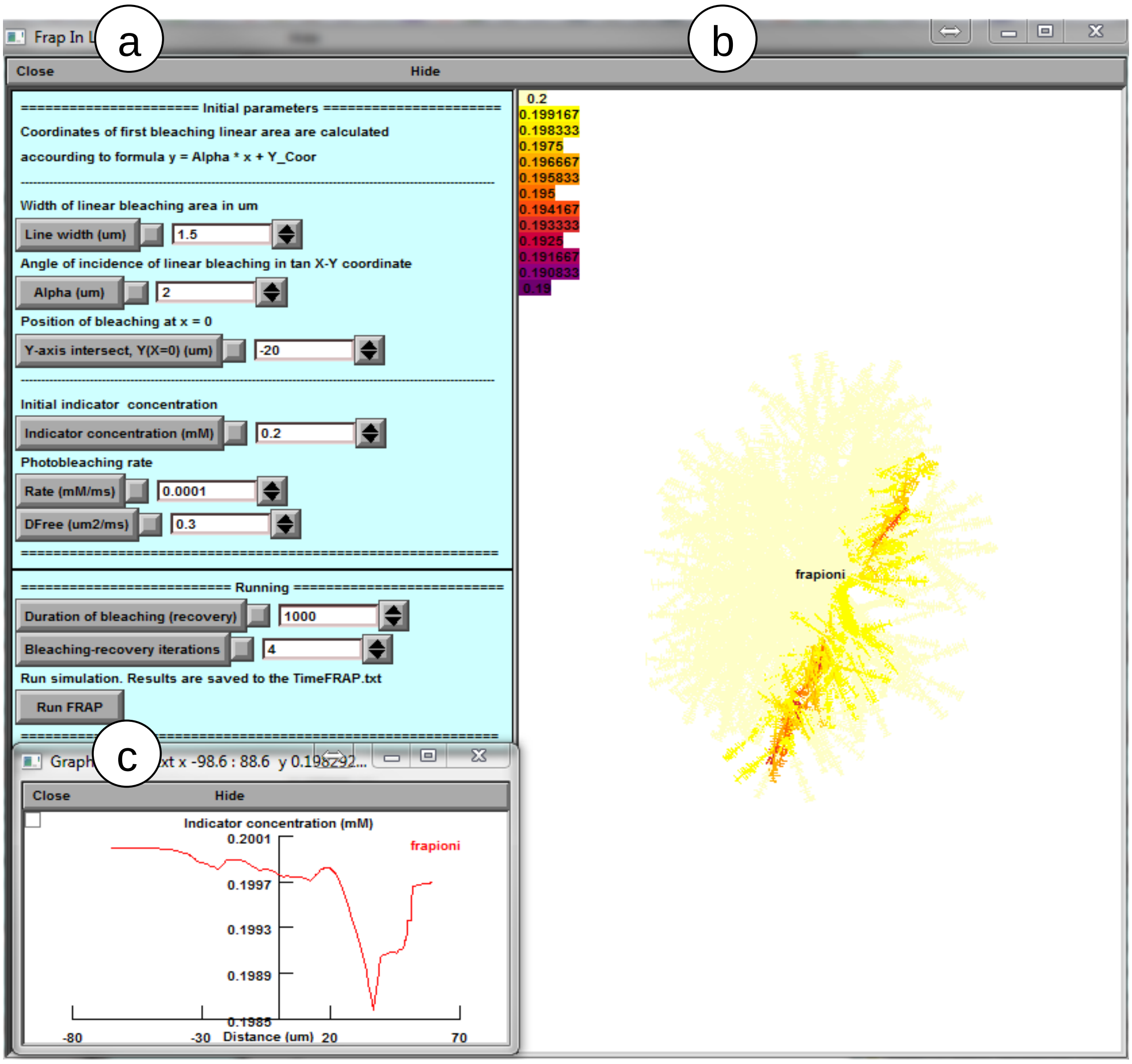

Figure 21. Window panels to control and monitor simulated linescan FRAP. a, Parameter settings panel. **b,** Concentration dynamics of non-bleached molecules, colour-coded and mapped on cell morphology. **c**, Concentration space profile sample.

The default duration of FRAP trials is 8000 ms, with photobleaching occurring every 1000 ms. In addition to the colour-coded shape map display (Fig. 21b), the results of FRAP simulations could be plotted and saved using standard NEURON functions: for instance by selecting and identifying the cell branch / area of interest ('dendrite' in NEURON format) using **PointProcessGroupManager,** and next configuring the output display and store using the **Graph** menu (Fig. 21c).

Probing membrane mechanisms of astroglia

This part of ASTRO deals with simulations of membrane mechanisms (passive electrical properties, voltage-dependent channels, ion exchange, receptor current, etc.) and intracellular processes (Ca^{2+} entry, buffering, diffusion, and removal mechanisms) acting in morphologically realistic astrocytes. Most of the programming routines involved are an inherent part of the NEURON environment, with the full guidance available in the NEURON documentation https://www.neuron.yale.edu/neuron/docs.

Therefore the sections below provide only brief information relevant to astroglial probing.

Membrane voltage landscape

In the main control panel (Fig. 18), pressing **'Compute the spatial voltage distribution'** opens window panels (Fig. 22) which report membrane voltage across selected cell processes upon a step current injection at the soma (a common electrophysiological test scenario). The default resting potential is -85 mV.

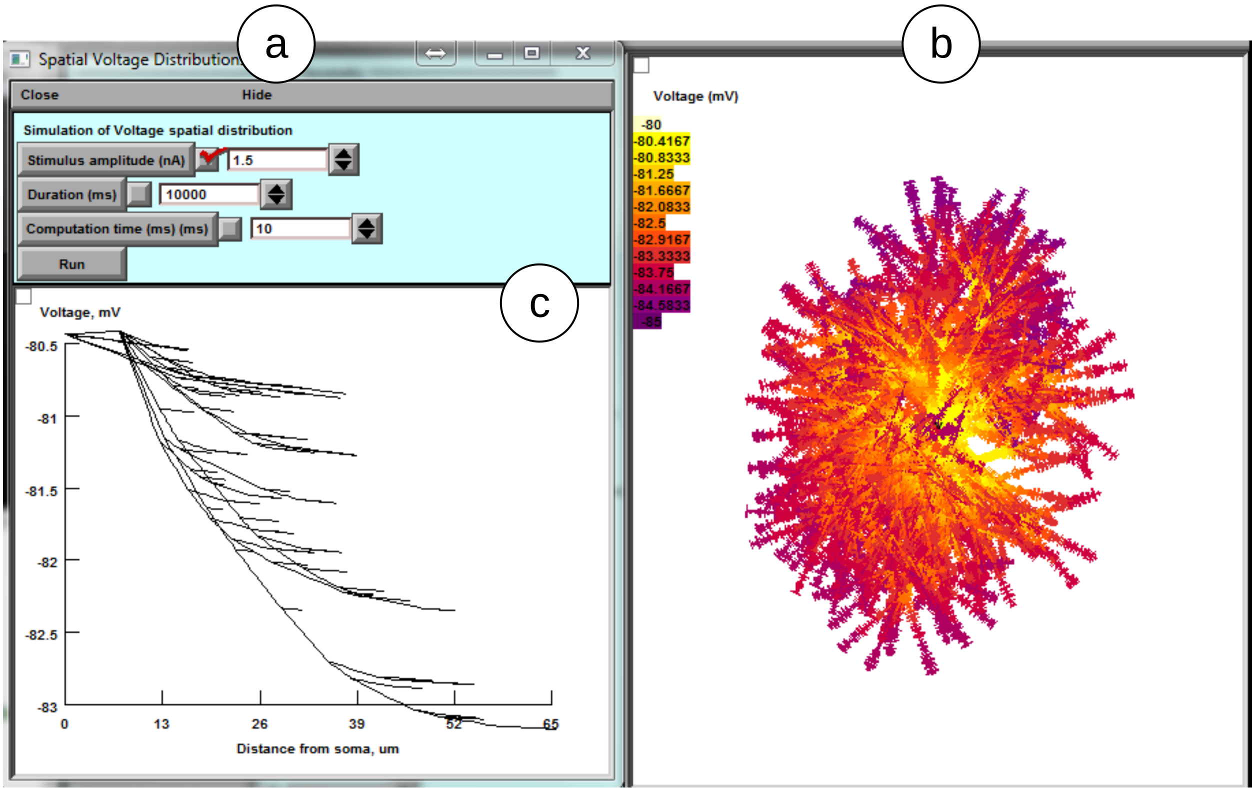

Figure 22. Window panel to monitor membrane voltage distribution including a gap junctions feature.

a, Settings panel. **b,** Dynamic voltage landscape mapped on the cell morphology. **c,** The corresponding voltage profile plot along individual processes ('dendrites' in NEURON).

The initiation panel (Fig. 22a) provides the following settings:

'Stimulus amplitude (nA)', amplitude of constant depolarising current injected into the soma.

'Duration (ms)', duration of constant depolarising current.

'Computation time (ms)', simulation run time (time of display).

'Run', to start simulation.

Simulating membrane voltage in response to local current hotspots

This ASTRO function can be performed by modifying settings in the ASTRO menu section below titled **'Simulating astroglial glutamate transporters'**. The latter provides full monitoring of the dynamic membrane voltage landscape mapped onto the cell morphology.

Gap junctions

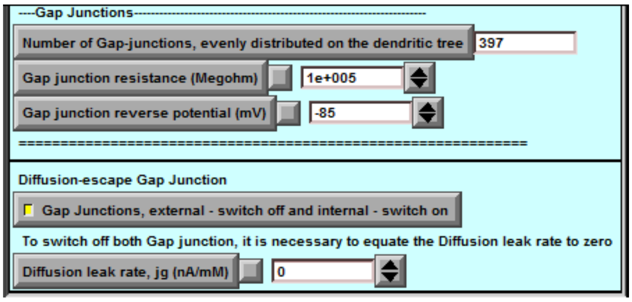

ASTRO has basic modelling provisions for the two types of gap junctions, which simulate the bulk diffuse (intracellular and extra cellular connections) and electrical connections between adjacent astroglia (control panel in Fig. 18a, bottom).

Gap junction menu (fragment of Fig. 18a, bottom).

The first type is a local electrical connection (current sink), which will affect local membrane potential V_m. It sets the local transmembrane current as $(V_g-V_m)/r_g$ where V_g and r_g are the gap junction reverse potential and resistance, respectively.

'Number of Gap junctions, evenly distributed on the dendritic tree': indicates the total number of gap junctions scattered uniformly across astrocyte processes ('dendrites' in NEURON terminology).

'Gap Junction Resistance r_g (MOhm)': a default value of 10^{10} MOhm, equivalent to having zero gap junction current.

'Gap Junction Reverse potential V_g (mV)': the default value is set at -85 mV.

The second type (**'Diffusion escape gap junction'**) is a local diffusion leak. It is determined by the rate of $j_g(C_g-C_0)$ where j_g, C_g, and C_0 are the leak constant, local intracellular ion/molecule concentration, and intracellular ion/molecule concentration on the other side of gap junction (resting concentration by default), respectively. The default value of j_g is zero, reflecting no gap junction diffusion leak.

'Diffusion leak rate, jg (nA/mM)': sets j_g value.

'Gap Junctions, external – switch off and internal – switch on': switches between inter-cellular (external) and internal gap junctions. The flux of internal (autaptic) gap junction is $j_g(C_g-C_{g1})$, where the C_{g1} is the ion concentration at the connected part of astrocyte ; the connecting compartment is currently set to be chosen randomly from the nanoscopic processes in the area; the internal gap

junction procedure is to be further adjusted in accord with additional further experimental constraints.

Modelling intracellular calcium dynamics

ASTRO provides simulation and analyses of the intracellular Ca^{2+} dynamics - including entry, buffering, diffusion, regenerative waves, and removal - across the entire astrocyte morphology. This type of simulation may require significant computational resources. Shorter-duration (1-1000 ms) trials could be feasible on the Host computer alone. Simulating longer events over large cell areas will probably require a dedicated Worker computer / cluster (see below for detail).

The current ASTRO version enables simulation of the two following scenarios: (i) macroscopic Ca^{2+} dynamics over the entire cell morphology, and (ii) oscillatory, single-channel type Ca^{2+} entry dynamics on the nanoscopic or microscopic scale within one or two selected processes.

Ca^{2+} wave simulations

Pressing **'Calcium wave'** key on the main control panel (Fig. 18c) opens new windows (Fig. 23) for setting, controlling, and displaying intracellular Ca^{2+} dynamics in response to a local increase in the concentration of the Ca^{2+} channel ligand IP_3.

ASTRO employs the standard NEURON-integrated mathematical formulism of Ca^{2+} reaction-diffusion kinetics including buffering and removal, which has been tested and validated in numerous studies; its detailed description can be found in the ***cadifus.mod*** file. The basic set of equations of calcium dynamics is as follows (built-in NEURON functions and forma are used).

$\frac{d[Ca]}{dt} = Dca\frac{d^2[Ca]}{dt^2} - \frac{\pi(i_{ca} - i_{pmp}) \times diam}{2 \times FARADAY}$, where $[Ca]$ is Ca^{2+} concentration, Dca diffusion coefficient, $diam$ is local diameter, i_{ca} is the sum of voltage dependent potassium currents, and the pump current i_{pmp} *is*

$i_{pmp} = 2\frac{FARADAY \times (f_{flux} - b_{flux})}{area}$, *where* f_{flux} *and* b_{flux} are the forward and reverse fluxes

Two types of Ca^{2+} buffers are used, mobile *bufm* and endogenous *bufs*,
with the corresponding kinetic constants *kf* and *KD*

$$\sim\ [Ca] + bufs \leftrightarrow cabufs\ \ (kfs,\ KDs \times kfs)$$

$$\sim\ [Ca] + bufm \leftrightarrow cabufm\ \ (kfm,\ KDm \times kfm)$$

SERCA pump, channel, and leak with alpha, relative abundance of SERCA mechanisms:

$$pump\ current: \frac{d[Ca]}{dt} = -v_{\max} \times \frac{[Ca]^2}{[Ca]^2 + Kp^2}$$

$channel :\sim\ hc \leftrightarrow ho\ \ (kon \times Kinh,\ kon \times ca),\ j_{chnl}$ is Ca^{2+} flux releases from SERCA to cytoplasm

$$\frac{d[Ca]}{dt} = alpha \times j_{\max} \times \left(1 - \frac{[Ca]}{caer}\right)\left(\frac{ip3i}{ip3i + Kip3} \times \frac{[Ca] \times ho}{ca + Kact}\right)^3$$

$leak: \frac{d[Ca]}{dt} = alpha \times L \times \left(1 - \frac{[Ca]}{caer}\right)$, where $L = v_{\max} \times [Ca]^2 \Big/ \left(\frac{[Ca]^2}{[Ca]^2 + Kp^2}\left(1 - \frac{[Ca]}{caer}\right)\right)$

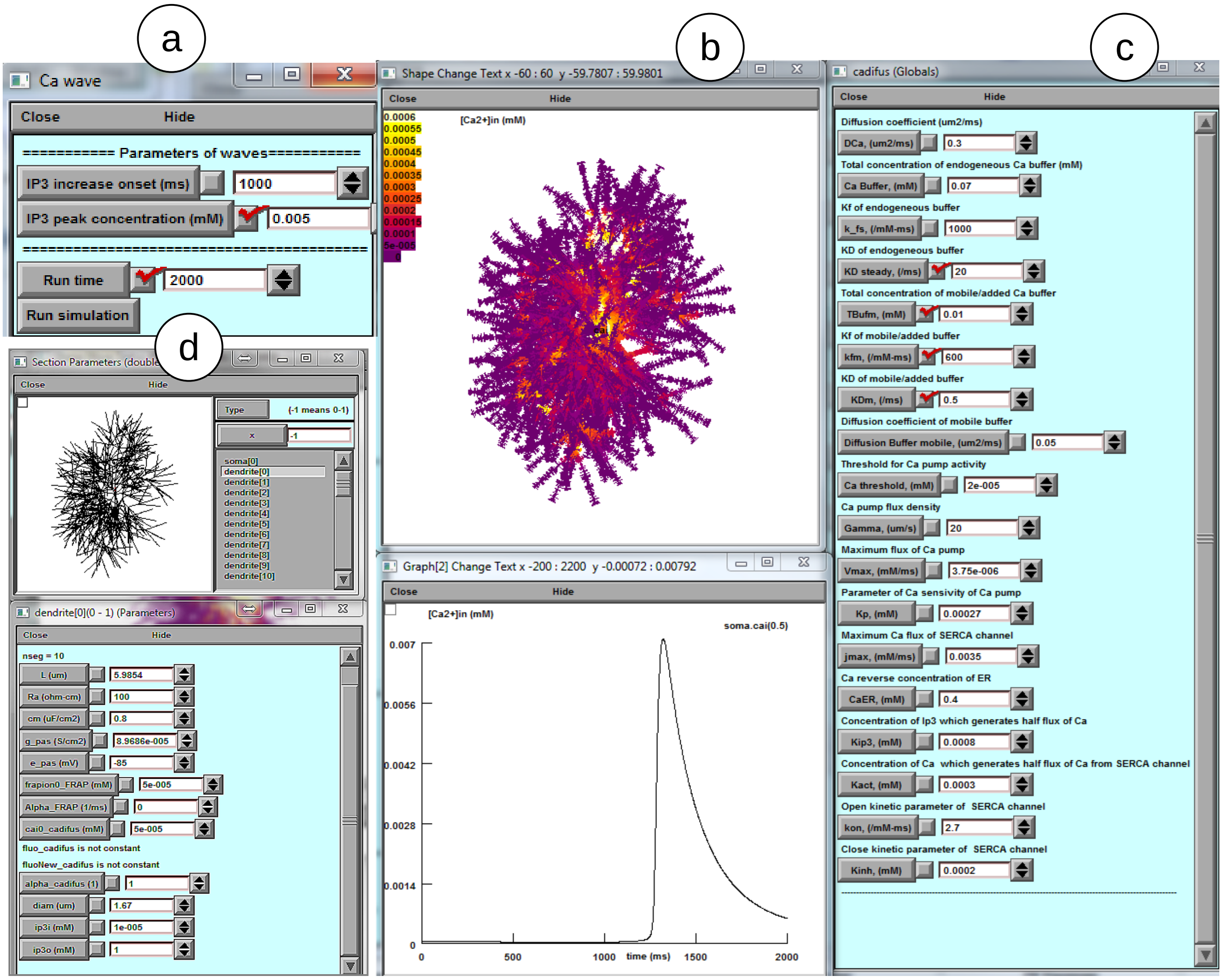

Figure 23. Window panels to control and monitor Ca^{2+} waves dynamics. a, Ca^{2+} wave trigger settings panel. **b**, Colour-coded, dynamic [Ca^{2+}] landscape mapped onto cell morphology (top) and readout plot of [Ca^{2+}] dynamics the soma (by default; bottom). **c**, Parameter setting panel for Ca^{2+} reaction-diffusion processes. **d,** Window panel to monitor Ca^{2+} homeostasis and dynamics at individual processes ('dendrites' in NEURON nomenclature).

'Ca wave' panel (Fig. 23a) has controls as follows:

> **'IP_3 increase onset (ms)'**, sets the onset of a step increase in the IP_3 concentration;
>
> **'IP_3 peak concentration (mM)'**, sets amplitude (0.005 mM shown).
>
> **'Run time'** sets simulation run time (default 2000 s).

The window parameter setting panel for Ca^{2+} reaction-diffusion (Fig. 23c) displays the corresponding explanations above the setting keys. Concentration and kinetic parameters of the Ca^{2+} indicator (Fluo-4) are constrained by experimental measurements whereas the default values for Ca^{2+} pumps and channels correspond to estimates found in the literature. These parameters should be further constrained by the user based on specific experimental tests.

A new simulation run can start once the current run has ended. **'Graph'** panel (Fig. 23b, bottom) provides [Ca^{2+}] time course in the soma. The current ASTRO version deals with IP_3 rises at the soma but it can be adapted to 'release' IP_3 locally at any place in the astrocyte.

Choosing between IP_3-dependent mechanisms of Ca^{2+} signalling

In addition to the NEURON-default IP_3-dependent Ca^{2+} mechanism, one can use two alternatives developed earlier specifically for astroglia: one incorporating glutamate-dependence of IP3 concentration (De Pitta et al, 2009, *J Biol Phys* 35:383-411), and one involving a specific IP3-receptor dynamics (Fink et al, 2000, *Biophys J* 79:163-83). To use the De Pitta mechanism, (i) set IP3 concentration to zero (Fig. 23a panel), and (ii) invoke ***cadifus*** mechanism in the standard '**MechType**' NEURON menu: glutamate concentration set by the '**ModelStim_cadifus**' button. To run the Fink model, (i) copy all mod-files from directory ...*Astro-master\neuronSims\ ExtraMechanism fink2000* to the main directory ...*Astro-master\neuronSims*, and (ii) compile these files in NEURON using the standard ***mknrndll.exe*** command.

Simulating microscopic Ca^{2+} events

Pressing **'Calcium dynamics'** key (Fig. 18c) prompts four new windows (Fig. 24):

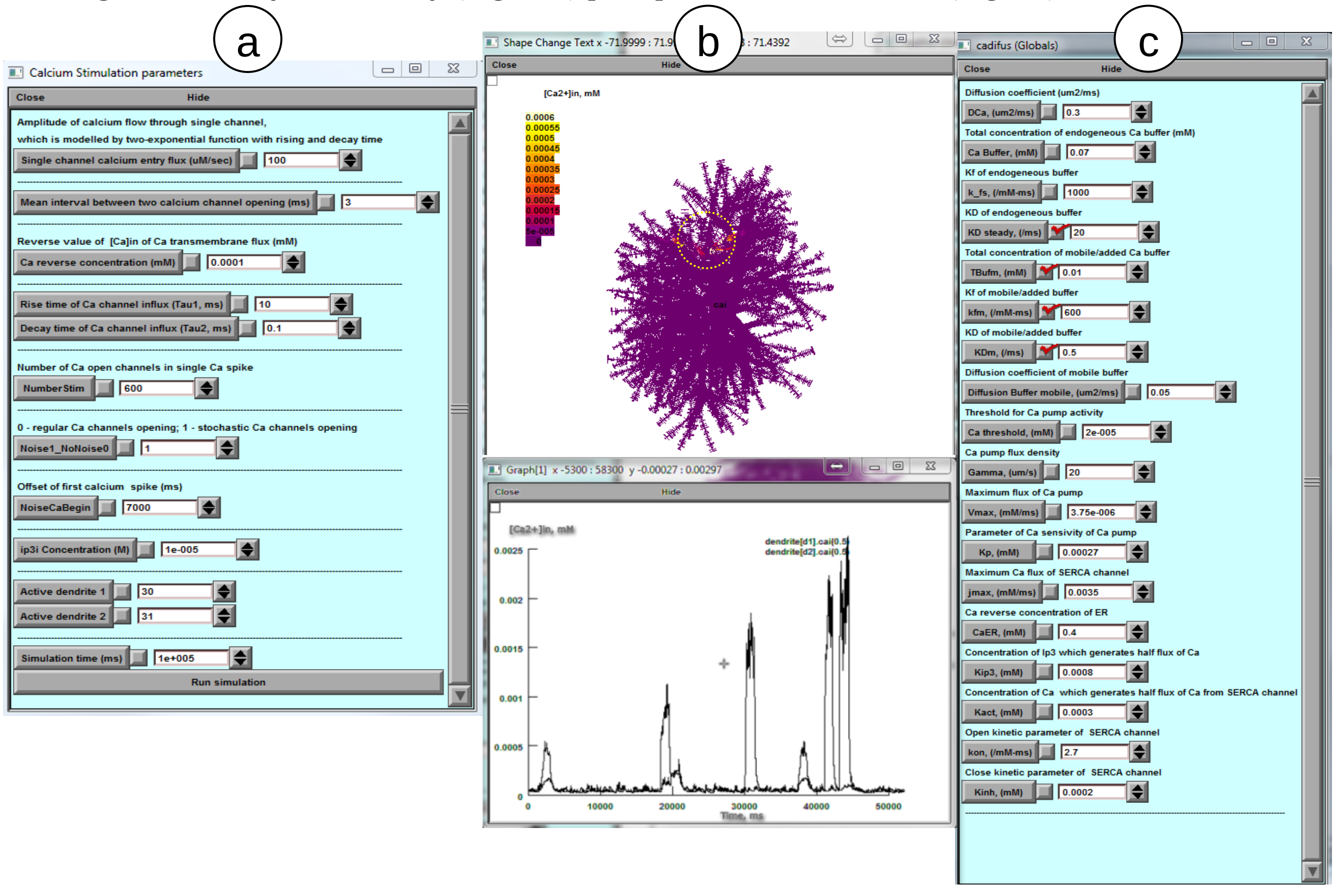

Figure 24. Window panels to control and monitor microscopic [Ca^{2+}] dynamics. a, Parameter settings for single-channel Ca^{2+} dynamics ('sparks' and 'puffs') in two selected 'active' branches (bottom: 'dendrite' numbers 30 and 31 are shown, in NEURON nomenclature). **b**, Visualisation of internal [Ca^{2+}] dynamics mapped on cell morphology (top), and [Ca^{2+}] time course in two selected dendrites (bottom; dendrites d1 and d2 are shown); yellow circle depicts the area of interest ('dendrites' 30 and 31). **c**, Parameter setting panel for Ca^{2+} reaction-diffusion processes (as in Fig. 23c).

Window panel **'Calcium stimulation parameters'** (Fig. 24a) provides parameter settings for microscopic Ca^{2+} entry kinetics. The latter is assumed to consist of a series of stochastic Ca^{2+} channel openings (individual events) including higher-frequency bursts. Note that many of the parameters

involved are currently unknown and therefore represent the subject of investigative exploration and probing.

'Single channel calcium entry flux' sets the rate of calcium entry during an individual event.
'Mean interval between two calcium events' is the average time interval between events.
'Basal Ca2+' sets is basal [Ca^{2+}] which is reverse concentration for the linear Ca^{2+} efflux.
'Rise' and **'Decay'** set the rise and decay time of Ca^{2+} flux during a single channels opening.
'Events per burst' sets the number of calcium channels opening per burst.
'Randomness' is set to 0 for uniformly random channel opening (Poisson process) with, or to 1 for cyclic channel opening.
'Burst onset' sets the time of the event burst.
'IP3 concentration' sets the initial concentration of IP3 ions.
'Active dendrite 1' and 'Active dendrite 2' are astroglial processes ('dendrites' in NEURON terminology) where active Ca^{2+} entry is enabled.
'Stimulation time' sets the run time of simulations. In astroglia, Ca^{2+} dynamics is a relatively slow process and the simulation trial normally requires needs at least 100 seconds.

Simulating astroglial glutamate transporters

Astroglial plasma membranes are enriched in high-affinity glutamate transporters which generate rapid inward current upon glutamate binding. In ASTRO main panel, pressing **'Glutamate transporters'** key (Fig. 18c, bottom) opens the menu (Fig. 25), which enables simulations of the dynamic membrane voltage landscape mapped onto cell morphology, in response to volume-limited application ('uncaging') of glutamate at a selected area of the cell.

'Uncaging glutamate' panel (Fig. 25a, top) provides self-explanatory parameter settings for volume-limited glutamate application (uncaging) within a round area of the cell. The *glutamate transporter kinetics* (Bergels and Jahr, 1997 Neuron 19: 1297-1308) includes six independent states (C_i, where i =1,…,6). The detailed description can be found the *GluTrans.mod* file. The basic relationships are

$$\frac{dC_1}{dt} = C_1[Glu]_o\ k_{12}\ u(v,\text{-}0.1) + C_2k_{21}$$
$$\frac{dC_2}{dt} = C_2[Na]_o\ k_{23}u(v,0.5) + C_3k_{32}$$
$$\frac{dC_3}{dt} = C_3k_{34}\ u(v,0.4) + C_4k_{43}$$
$$\frac{dC_4}{dt} = C_4k_{45} + C_5k_{54}[Glu]_{in}$$
$$\frac{dC_5}{dt} = C_5k_{56}\ u(v,0.6) + C_6\ k_{65}[Na]_{in}$$
$$\frac{dC_6}{dt} = C_6[K]_{in}k_{61} + C_1k_{16}u(v,0.6)[K]_o$$

Parameters

k_{12}	= 20	$(mM\ ms)^{-1}$	k_{21}	= 0.1	ms^{-1}
k_{23}	= 0.015	$(mM\ ms)^{-1}$	k_{32}	= 0.5	ms^{-1}
k_{34}	= 0.2	ms^{-1}	k_{43}	= 0.6	ms^{-1}
k_{45}	= 4	ms^{-1}	k_{54}	= 10	$(mM\ ms)^{-1}$
k_{56}	= 1	ms^{-1}	k_{65}	= 0.1	$(mM\ ms)^{-1}$
k_{16}	= 0.0016	$(mM\ ms)^{-1}$	k_{61}	= 2×10^{-4}	$(mM\ ms)^{-1}$
$[Na^+]_{in}$	= 15	mM	$[Na^+]o$	= 150	mM
$[K^+]_{in}$	= 120	mM	$[K^+]o$	= 3	mM
$[Glu^-]_{in}$	= 0.3	mM	$[Glu^-]o$	= 20×10^{-6}	mM

$$where\ \ u(th,x) = \exp(th \times x/(2 \times 26.7))$$

NOTE: The onset of uncaging must be at t > 3 ms, to ensure stable membrane kinetics. The user can add another, simultaneously occurring uncaging spot specifying the distance between the two spots at the distance X from the first one.

Transporter currents are computed in somatic voltage clamp configuration: the clamp parameters are indicated on the panel **'Somatic voltage clamp'** (Fig. 25b) including electrode resistance, clamp voltage and duration. The monitoring panel (Fig. 25c) display colour-coded membrane current landscape mapped onto cell morphology; the graph panel (Fig. 25d) displays the time course of extracellular glutamate and clamp current at selected cell processes.

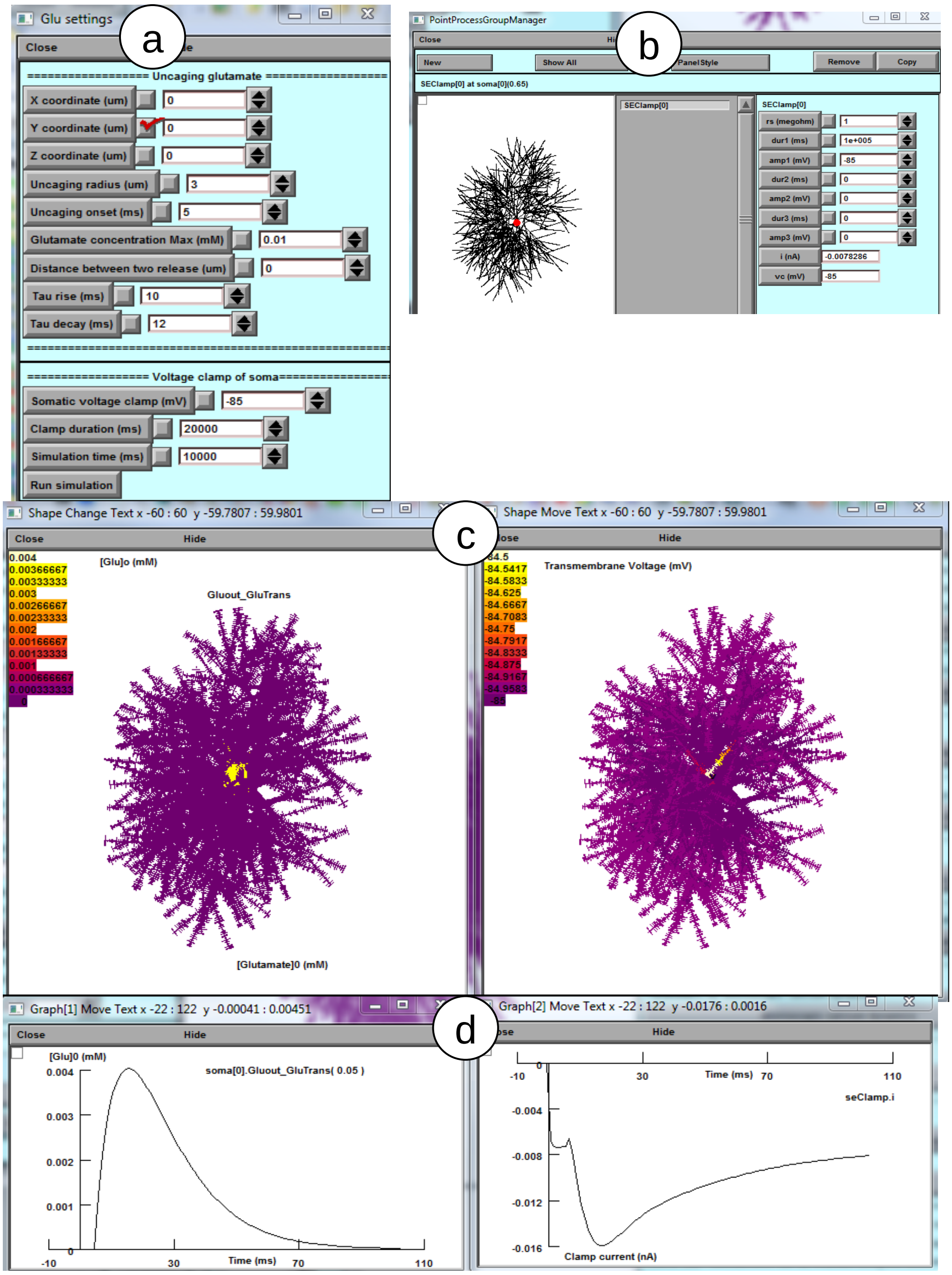

Figure 25. Window panels with parameter settings and readout plots, to simulate the dynamic membrane voltage landscape mapped onto cell morphology, in response to volume-limited application ('uncaging') of glutamate. a, Glutamate uncaging parameter settings. **b,** Voltage-clamp parameter settings including electrode positioning. **c,** Visualisation panel. **d,** Time course graph panel.

Window panel **'Glu setting'** (Fig. 24a) provides parameters:

'X, Y and Z coordinate (um) ' sets the centroid coordinates for the uncaging spot.
'Uncaging radius (um) ' sets the uncaging spot radius.
'Uncaging onset (ms) ' sets the onset of uncaging.
'Glutamate concentration Max (mM)' sets the maximum glutamate concentration during uncaging.
'Rise' and 'Decay tau (ms)' set the dynamics of glutamate uncaging.

Simulating potassium dynamics inside and outside astroglia

This option is to simulate the dynamics of intracellular [K^+] resulting from local K^+ input and intracellular redistribution and efflux / leakage (Fig. 26). From the main panel of ASTRO (Fig. 18) the user can activate the potassium dynamics by clicking the button **'Potassium Dynamics'** (Fig. 18c, bottom) which prompts the relevant windows (Fig. 26).

A standard NEURON panel (Fig. 26a) is to control simulation and visualise the outcome. The **'Potassium settings'** panel (Fig. 26b) provides self-explanatory parameter settings for volume-limited potassium application (local current) within a round area of the cell. Here, [K^+] dynamics are computed in somatic voltage clamp configuration: the clamp parameters are indicated on the panel **'Somatic voltage clamp'** (Fig. 26a, bottom) including electrode resistance, clamp voltage and duration. Intracellular potassium buffering is modelled with the linear approximation current

$$I_K = K_p \left(\frac{[K^+]_{in}}{[K^+]^0_{in}} - 1 \right),$$ where

$[K^+]_{in}$ and $[K^+]^0_{in}$ denotes, respectively, the present and basal intracellular K^+ concentrations, K_p (mA/cm^2) is the pump rate.

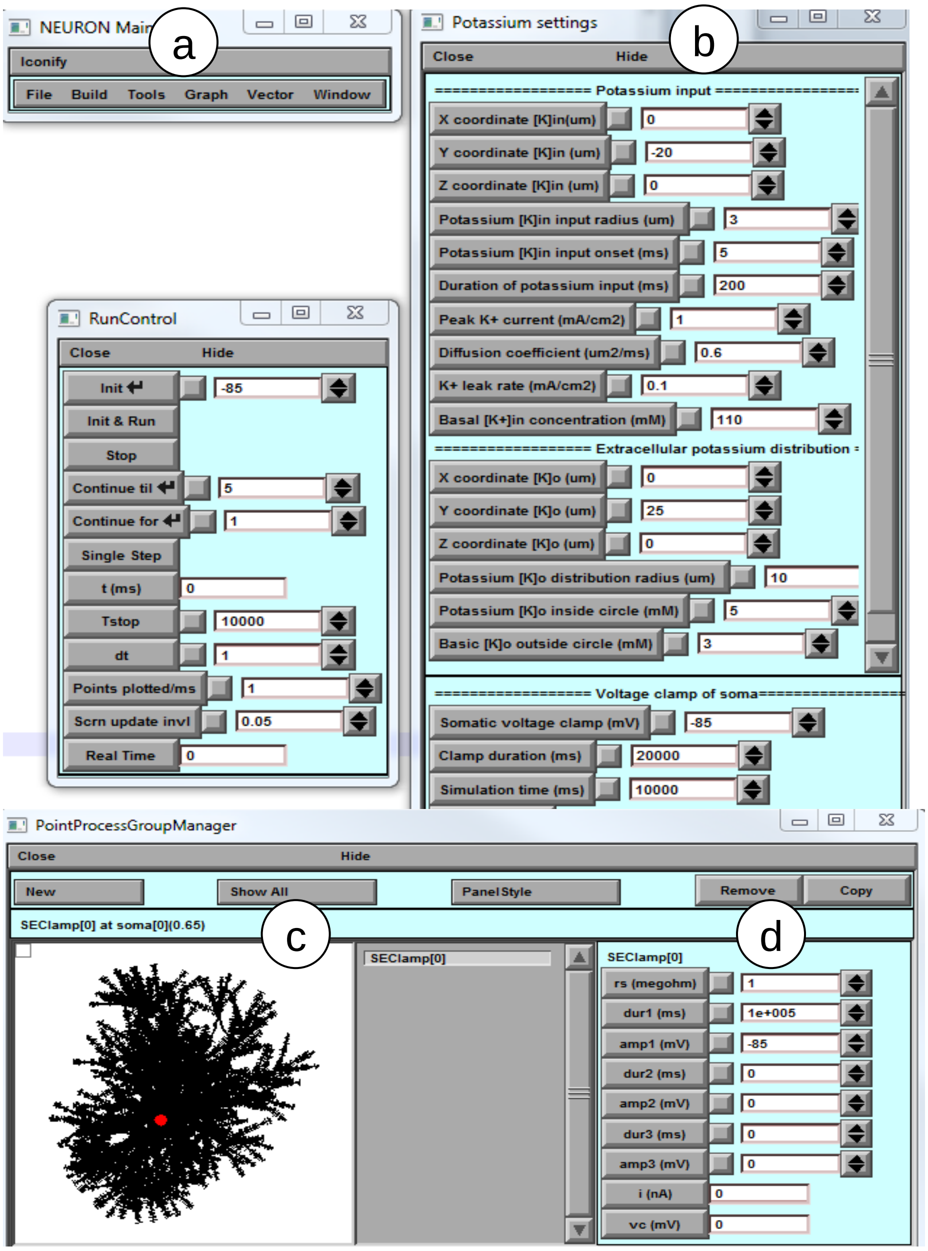

Figure 26. Window panels to control and monitor intracellular K^+ dynamics and extracellular K^+ steady-state distribution. a, Parameter settings for monitoring [K^+]$_{in}$ and [K^+]$_{out}$ landscape mapped onto cell morphology. **b,** Parameter settings pertinent to volume-limited [K^+]$_{in}$ entry and [K^+]$_o$ distribution. **c,** Shape visualisation with voltage-clamp positioning. **d,** Somatic voltage clamp

applied (bottom), for the sake of simplicity.

NOTE: The present simulation feature explores the diffusion landscape of intracellular K^+ while approximating the K^+ entry and extrusion kinetics with first-order reactions that are independent of membrane voltage, for the sake of clarity. A more detailed simulation paradigm could include further experimental constrains based on known (voltage- and concentration-dependent) K^+ pumps and channels, extracellular K^+ dynamics, and unclamped membrane voltage. Such membrane mechanisms, including astroglial K_{ir}4.1 channels, are available through the standard NEURON menus.

The monitoring panel (Fig. 27a) displays colour-coded $[K^+]_{in}$ concentration landscapes mapped onto cell morphology. The corresponding plot (Fig. 27c) shows the time course of intracellular $[K^+]_{in}$ at a selected location (dendrite [51]). The panel (Fig.27b) shows the steady state distribution of extracellular $[K^+]$ onto cell morphology.

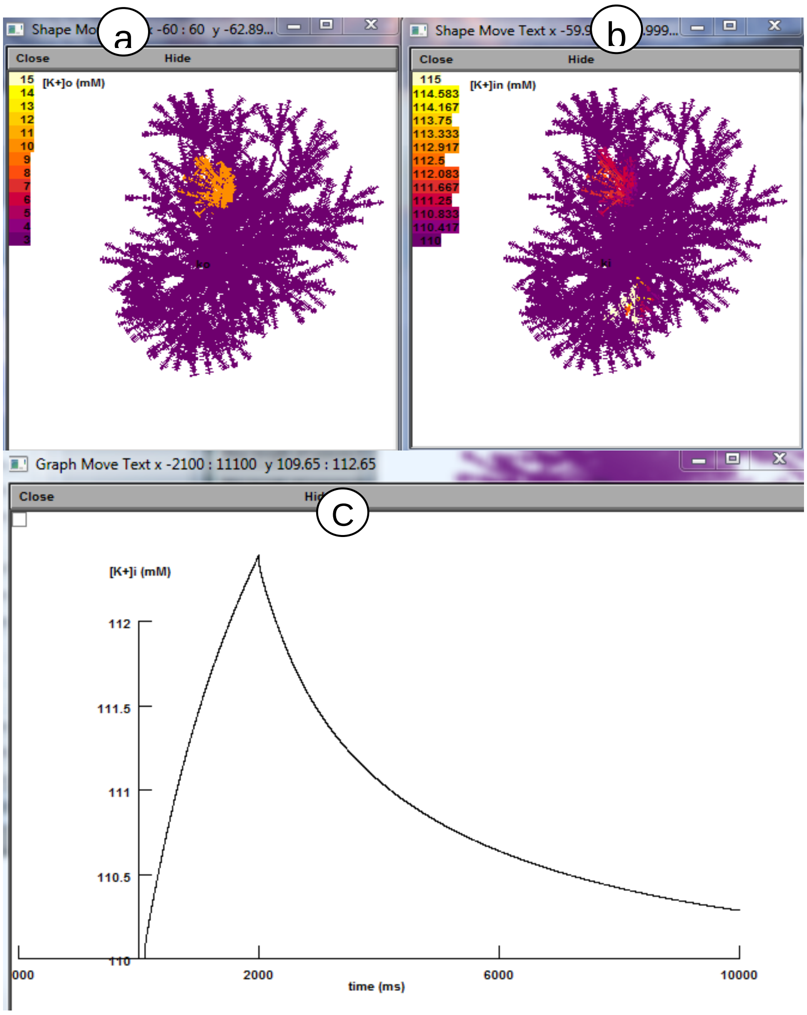

Figure 27. Window panels displaying simulated dynamics of intracellular $[K^+]_{in}$ (a) and extracellular $[K^+]_{out}$ (b) mapped onto cell morphology, and of the $[K^+]_{in}$ time course (c) at a selected branch (dendrite), in response to volume-limited injection of potassium current (as in Fig. 26).

Window panel **'Potassium setting'** provides parameter settings

'X, Y and Z coordinate [K]in (um)' sets centroid co-coordinates for the K^+ entry area.
'Potassium [K]in input radius (um) ' sets the radius of K^+ entry area.
'Potassium [K]in input onset (ms)' sets the onset of K^+ entry.
'Duration of potassium input (ms)' sets the duration of K^+ entry.
'Peak K+ current (mA/cm2)' sets maximum amplitude of K^+ current entry.
'Diffusion coefficient (um2/ms)' sets the intracellular K^+ diffusion coefficient.
'K+ leak rate (mA/cm2)' sets K_p, the K^+ extrusion current density.
'Basal [K+]in concentration (mM)' sets the resting intracellular $[K^+]$.

Window panel “**Extracellular potassium distribution**”:
'X, Y and Z coordinate [K]o (um) ' sets the centroid co-coordinates for the extracellular region of the $[K^+]$ fluctuation.

'Potassium [K]o distribution radius (um)' sets the radius of the extracellular K^+ region.
'Potassium [K]o inside circle (mM)' sets extracellular $[K^+]$ inside the region.
'Basic [K]o outside circle (mM)' sets extracellular $[K^+]$ outside the region.

SYSTEM PREPARATIONS FOR HIGH-END CALCIUM SIMULATIONS

Preparing Worker computer / cluster (HPC, OS Linux) for Ca^{2+} simulations

1. The remote cluster for ASTRO computations should be prepared only once. Subsequent computations can be carried out using the host computer only. In rare cases, when the cluster part of ASTRO requires substantial modifications the user will have to make changes using C++ and HOC code in HPC directory.
2. Installing an ASTRO kernel on the cluster requires the "mpic++" (***Message Passing Interface***) compiler present in the system path. The free version of mpic++ can be found here https://www.open-mpi.org/software/ompi/v2.0/. Most clusters operating under Linux have this compiler in the system by default.
3. Download the folder HPC with all its content from https://github.com/LeonidSavtchenko/ASTRO. This folder must be saved to the place shared between cluster nodes, for instance, the directory "/home/<***username***>". Sharing files between the host computer and the cluster can be achieved using free software "WinSCP" https://winscp.net/eng/download.php.
4. Install NEURON on the cluster. The latest version can be downloaded from the official site. We recommend using an installation from a source code from here https://www.neuron.yale.edu/neuron/download/getstd and following steps 1-5 of the relevant instruction https://www.neuron.yale.edu/neuron/download/compile_linux

   (**--with-paranrn** option has to be added to the configure command for NEURON installation to enable distributed computations).

   NEURON GUI is not generally required for cluster simulations. To remove it from the installation, do not download *iv-mm.tar.gz* archive and replace --*with-iv=$HOME/neuron/iv* with --*without-iv* when calling *configure* for NEURON installation.

The free-download PuTTY and WinSCP programs can be used to work with the console and file system of the remote cluster.

The structure of the directors, in GitHub, necessary for working with the cluster has the form:

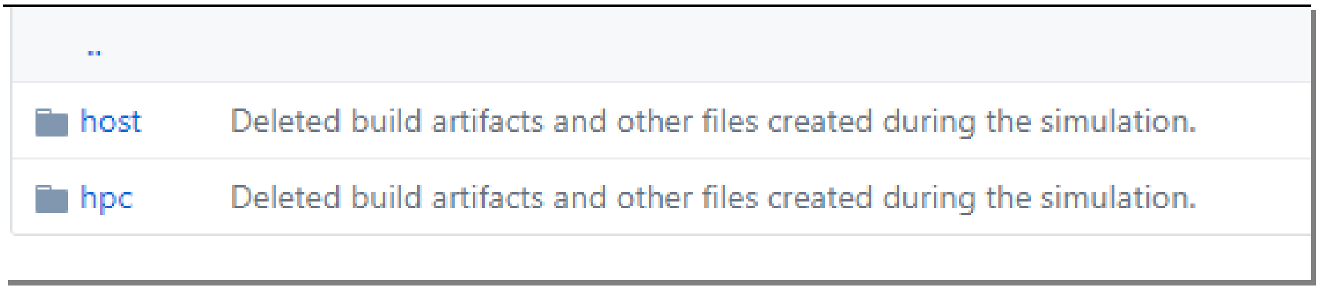

NOTE: All files from directory HPC should be downloaded on the cluster, keeping the structure of directories unchanged.

Preparing Host computer (client, OS Windows) for Ca^{2+} simulation

1. Open *clusterCaSim/host/scripts/win-lin/params.bat* and set your cluster connection parameters including the path to the *hpc* folder located in the cluster. Below is the corresponding fragment of the ***params.bat*** file.

   ```
   *****************************************
   set HEADNODEIP=144.82.46.83
   set LOGIN=my_login
   set PASSWORD=my_password
   set HEADNODEWORKERDIR=/home/*******/hpc
   *****************************************
   ```

2. Open ***clusterCaSim/host/core/BasicParams.m*** and add "avaliableNodes" variable with names of the user cluster nodes.

   The corresponding code fragment:

   ```
   % Whether to conduct simulation on remote HPC cluster
      remoteHPC = true;

      % Whether to ZIP I/O data files before transferring through a network
      zipDataFiles = false;

      defaultGeometry = 'default - AstroGeometry';

      % Names of all cluster nodes you may want to use in the simulation
      % (must be kept in sync with those ones defined in "hpc\hostfile_BusyMaster and
   hpc\hostfile_IdleMaster")
      availableNodes = {'tuxmaster', ...
   ```

   Also, fill ***clusterCaSim/hpc/hostfile_BusyMaster*** and ***hostfile_IdleMaster*** files with node names in the following manner: each line should contain the name of the node followed by ' max_slots=1' without quotes.

   Contents of both files should be the same except that ***hostfile_IdleMaster*** should not include the master node (only slaves).

3. After launching ***start.m*** and pressing the key "Calcium dynamics on cluster" the user will have two options:

```
Please specify what to do:
1. Download and analyse results.
2. Start simulation from scratch (current results will be lost).
[1 or 2]: >>
```

a. Option 1. The user can upload from the cluster and analyse the previously obtained results.

b. Option 2. The user can start a new simulation.

Upon pressing Option 2 the following window appears

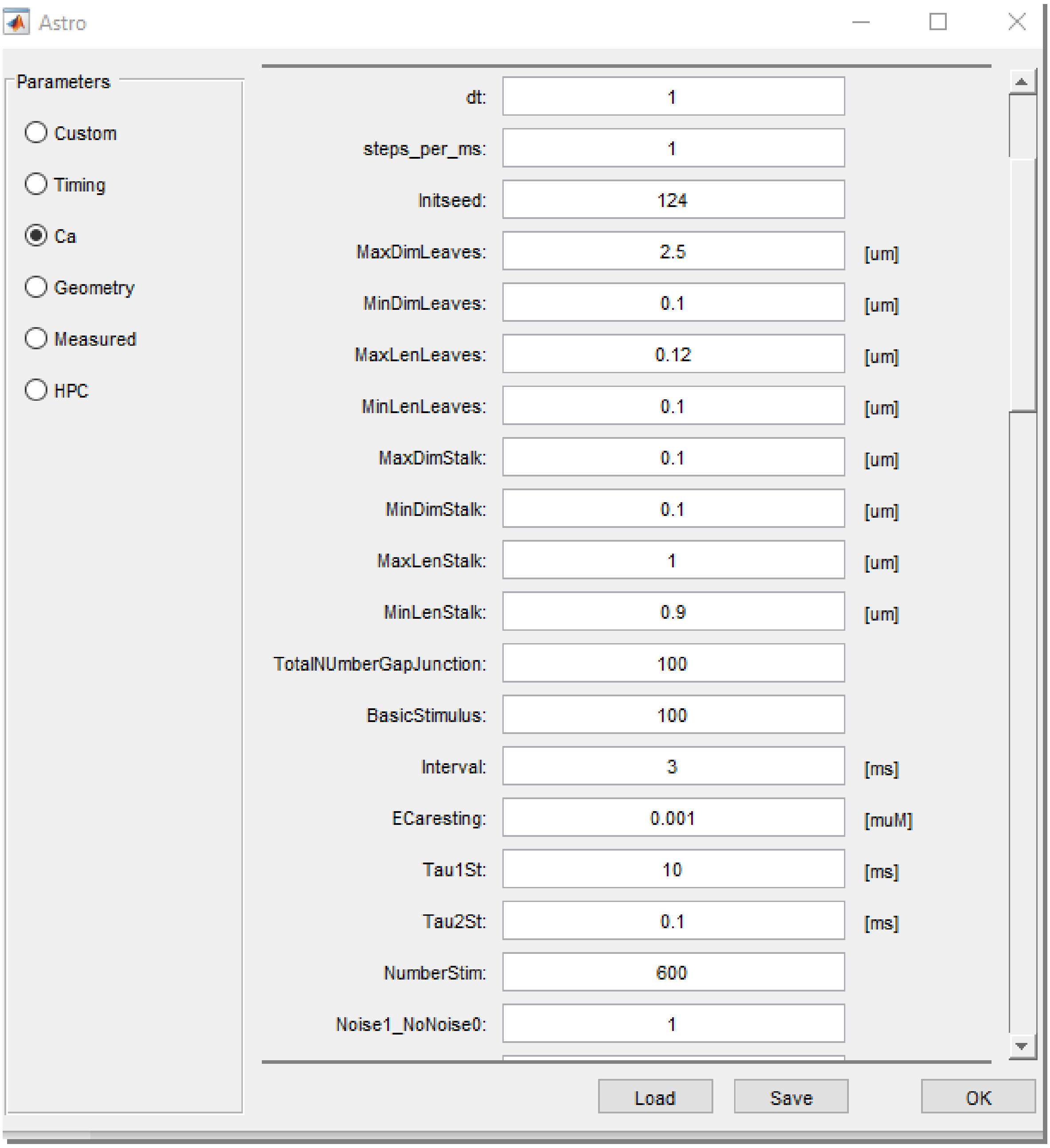

With this panel, the user can specify

1. Parameters of simulations (*dt* – time step of computation, step-per-ms – time step of data visualisation and InitSeed – the seed of random number generator).
2. Parameters of astrocyte Nano geometry.
3. Parameters of calcium dynamics.

The parameter definition here is the same as that for the astrocyte model on the local computer (also see Figs. 18, 23 and 24).

Astrocyte geometry can be uploaded onto the cluster using the structure of *hoc*-files, the same structure of files as for the geometry of ASTRO described previously (see part: ***Generating / downloading astrocyte stem tree)***.

The default astrocyte geometry is provided by the file ***AstroGeometry.hoc***.

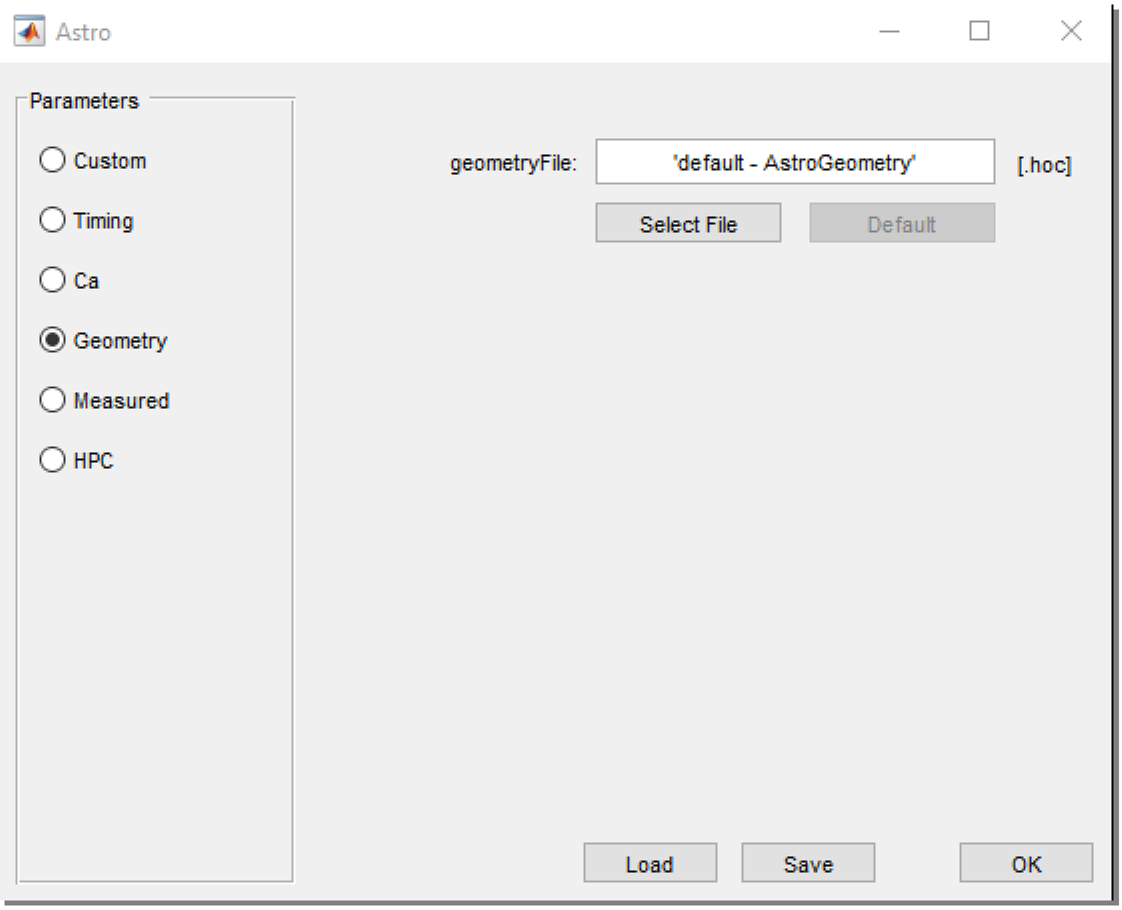

Parameters on the *HPC* cluster can be modified using the panel below:

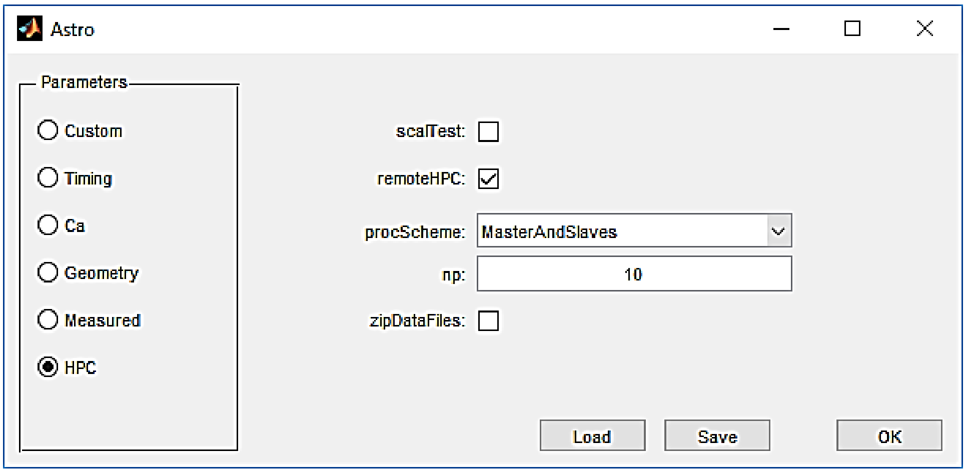

This panel provides three options:

scalTest – check this to have the scalability test. This test will show how well the execution time scales relative to the number of processes (see the setting **np**).

remoteHpc – uncheck this if ASTRO is to be run on the client PC only, without a cluster. The user should also modify NRNDIR and HPCDIR parameters that point to the NEURON and *hpc* folder locations on client PC in the *clusterCaSim/host/scripts/win-win/params.bat* and *params.sh*.

procScheme – processor distribution scheme. This parameter controls engagement of master and/or slave computers.

np – the number of processors for any slave computer.

Having changed any parameters in Matlab files, the user should recompile the executables using ***clusterCaSim/host/BUILD_AllHostExecutables.m***.

Technical Notes

Nano geometry

In data sets comprising 3D-reconstructed nanoscopic processes, all serial sections should be represented by (10-20) XYZ points scattered along the part circumvent, for diffusion simulations to work correctly.

Because of a significant amount of computations in complex diffusion simulation MATLAB cannot handle stopping or restarting it by pressing a button.

Calcium dynamics (Cluster)

There could be cases when certain selected geometry cannot be split into the specified number of processors. In this case, the user will see an MPI error before the computations begin. To solve the problem, the user can directly increase or decrease the number of processors.

Dendrites (astroglial processes will be called dendrites in the original NEURON environment) should be connected to the soma only in the 1 position. Otherwise cell splitting fails. Examples:

Correct: soma[0] connect dendrite[125](0), 1
Wrong: soma[0] connect dendrite[125](0), 0.5
Wrong: soma[0] connect dendrite[125](0), 0.1
Wrong: soma[0] connect dendrite[125](0), 0

This version of ASTRO User Guide assumes that the user will work on an in-house computer cluster. The future ASTRO versions will include a pre-installed package with the Amazon Cloud Computing services for general usage. Updates will be published on GitHub/LeonidSavtchenko/Astro.

SUPPLEMENTARY REFERENCES

1. Medvedev, N.*, et al.* Glia selectively approach synapses on thin dendritic spines. *Philos Trans R Soc Lond B Biol Sci* **369** (2014).
2. Rusakov, D.A. Disentangling calcium-driven astrocyte physiology. *Nature Rev. Neurosci.* **16**, 226-233 (2015).
3. Seifert, G.*, et al.* Analysis of astroglial K+ channel expression in the developing hippocampus reveals a predominant role of the Kir4.1 subunit. *J Neurosci* **29**, 7474-7488 (2009).
4. Sibille, J., Dao Duc, K., Holcman, D. & Rouach, N. The neuroglial potassium cycle during neurotransmission: role of Kir4.1 channels. *PLoS Comput Biol* **11**, e1004137 (2015).
5. Bergles, D.E., Tzingounis, A.V. & Jahr, C.E. Comparison of coupled and uncoupled currents during glutamate uptake by GLT-1 transporters. *J Neurosci* **22**, 10153-10162 (2002).
