# Supplementary material for: Disentangling astroglial physiology with a realistic cell model in silico
Source: Nat Commun. 2018 Sep 3;9:3554. doi: 10.1038/s41467-018-05896-w (PMC6120909; doi:10.1038/s41467-018-05896-w)
Supplement: Supplementary file 3 — Description of Additional Supplementary Files [file 41467_2018_5896_MOESM3_ESM.pdf]

Description of Additional Supplementary Files

File Name: Supplementary Movie 1
Description: A Monte Carlo test to compare electrodynamic properties of 3D-EM reconstructed and NEURON-compatible shapes of nanoscopic processes (related to Fig. 2f).

File Name: Supplementary Movie 2
Description: Illustration of a FRAP experiment in silico (related to Fig. 3e).

File Name: Supplementary Movie 3
Description: Current-voltage landscapes in the astrocyte membrane during GLT-1 transporter current triggered by spot-uncaging of extracellular glutamate (related to Fig. 4f).

File Name: Supplementary Movie 4
Description: Simulated spatiotemporal dynamics of a Ca^{2+} wave propagating inside astroglia (related to Fig. 6a).

File Name: Supplementary Movie 5
Description: Ca^{2+} activity of somatosensory cortex astroglia imaged with the GfaABC1D-LckGCaMP6f indicator in anaesthetised animals (related to Fig. 6c). Frame width 240 µm.

File Name: Supplementary Movie 6
Description: Ca^{2+} activity of somatosensory cortex astroglia imaged with the GfaABC1D-LckGCaMP6f indicator in awake animals (related to Fig. 6c). Red dots indicate Ca^{2+} waves which appear to occur in individual astroglia. Prominent, region-wide synchronous Ca^{2+} rises are likely to reflect motor/sensor activity. Frame width 240 µm.

File Name: Supplementary Movie 7
Description: Ca^{2+} activity of hippocampal astroglia (area CA1) imaged with whole-cell dialysed Fluo-4 in acute slices (related to Fig. 7a). Frame width 84 µm.

File Name: Supplementary Movie 8
Description: Simulated local Ca^{2+} activity based on Ca^{2+} spark mechanisms placed in several selected astrocyte braches (indicated in Fig. 7c), with Fluo-4 'added' to the cell *in silico* (related to Fig. 7c).
